# Implementation of the International Classification of Functioning, Disability, and Health (ICF) Core Sets for Children and Youth with Cerebral Palsy: Global Initiatives Promoting Optimal Functioning

**DOI:** 10.3390/ijerph15091899

**Published:** 2018-09-01

**Authors:** Verónica Schiariti, Egmar Longo, Alexander Shoshmin, Ludmila Kozhushko, Yanina Besstrashnova, Maria Król, Taynah Neri Correia Campos, Haryelle Náryma Confessor Ferreira, Cláudia Verissimo, Daniel Shaba, Matilda Mwale, Sandra Amado

**Affiliations:** 1 Division of Medical Sciences, University of Victoria, Victoria, BC V8W 2Y2, Canada; 2 Postgraduate Program in Collective Health, Faculty of Health Sciences of Trairi (UFRN-FACISA), Federal University of Rio Grande do Norte, Santa Cruz 59200-000, Brazil; egmarlongo@yahoo.es (E.L.); taynahneri@hotmail.com (T.N.C.C.); haryelle_naryma@yahoo.com.br (H.N.C.F.); 3 Federal Scientific Center of Rehabilitation of the Disabled named after G.A. Albrecht, Saint Petersburg 195067, Russia; shoshminav@mail.ru (A.S.); l.timch@mail.ru (L.K.); besstjan@mail.ru (Y.B.); 4 The Step by Step Association for Help of Disabled Children, Zamość 22-400, Poland; marikrol@poczta.onet.pl; 5 Health School Science, Polytechnic Institute of Leiria, Leiria 2411-901, Portugal; verissimo.cclaudia@gmail.com (C.V.); sandramado@gmail.com (S.A.); 6 RIPPLE Africa, Limbe 431136, Malawi, Africa; dan@rippleafrica.org (D.S.); mwalematilda81@gmail.com (M.M.); 7 Department of Education, Catholic University of Malawi, Limbe 431136, Malawi, Africa

**Keywords:** ICF Core Sets, child, cerebral palsy, disability, functioning, abilities, global health, service provision

## Abstract

*Background:* The International Classification of Functioning, Disability, and Health (ICF) Core Sets for children and youth with cerebral palsy (CP) offer service providers and stakeholders a specific framework to explore functioning and disability for assessment, treatment, evaluation, and policy purposes in a global context. *Objective:* Describe global initiatives applying the ICF Core Sets for children and youth with CP, with a focus on contributions to clinical practice and challenges in their implementation. *Methods:* This is a descriptive cross-sectional study. Ongoing initiatives applying the ICF Core Sets for CP in Russia, Poland, Malawi, and Brazil are included. *Results:* The main contributions of applying the ICF Core Sets for children and youth with CP include: (1) an objective description of abilities and limitations in everyday activities; (2) a consistent identification of facilitators and barriers influencing functioning; (3) a practical communication tool promoting client-centered care and multidisciplinary teamwork; and, (4) a useful guideline for measurement selection. The main challenges of adopting the ICF Core Sets are related to lack of ICF knowledge requiring intense training and translating results from standardized measures into the ICF qualifiers in a consistent way. *Conclusions:* Global initiatives include research and clinical applications at the program, service and system levels. The ICF Core Sets for CP are useful tools to guide service provision and build profiles of functioning and disability. Global interprofessional collaboration, capacity training, and informatics (e-records) will maximize their applications and accelerate adoption.

## 1. Introduction

Cerebral palsy (CP) is a lifelong condition that is caused by non-progressive damage to the infant brain [1], impacting the developmental trajectory of children, as well as their families [2,3,4,5]. CP is the most common physical disability in childhood. CP has a prevalence of approximately one in 500 live births, it is estimated that globally 17 million people have CP [6]. The overall prevalence of CP in high-income countries is 2.11 per 1000 live births [7], and 2.0 to 2.8 per 1000 live births in low- and middle-income countries [8]. In industrialized countries, the causative factors of the majority of cases of CP could not be identified and it has been postulated that many factors are likely to be prenatal in origin [9]. In resource poor countries, it has been postulated that there might be many potentially preventable factors causing CP, including asphyxia, meningitis, and cerebral malaria [8]. The diagnosis of CP relies on clinical suspicious and standardized motor assessments in a child who is not reaching motor milestones. In most of the cases, CP can be diagnosed in the first 12–18 months. Early and accurate diagnosis of CP is crucial for ensuring access to early intervention to optimize developmental outcomes [10]. Currently, there no known cure.

The clinical presentation of CP varies depending on the location and extend of the brain insult. In high-income countries, two in three individuals with cerebral palsy will walk, three in four will talk, and one in two will have social-cognitive abilities along the ability level of their peers [11]. Clinical symptoms associated with CP goes beyond the commonly observed in the musculoskeletal system, individuals with CP might present associated challenges in communication, learning, sensory, cognition, and behaviour, as well as seizure disorder. Due to the complex clinical presentation, individuals with CP and their families require coordinated support from health, education, and social services with significant economic burden [12]. However, regardless of the diagnosis and clinical presentation, children and young people with CP acknowledge that they have many *abilities*, and most importantly; with the right adaptations and modifications of the environment they can fully participate in activities of daily living [13,14].

Once the diagnosis of CP is ascertained or highly suspected, there are numerous tools to assess the impact of CP—from proxy or self-report perspectives—on different health-related domains, such as physical functioning, daily activities, quality of life, health-related quality of life, family well-being, education, and so on [15,16,17,18]. Moreover, there are several CP-specific interventions available to minimize or prevent secondary impairments and to promote reaching the individual’s functional potential and family well-being [11,19]. Given the different approaches to assessment and treatment for individuals with CP, lack of consensus on data collection, and data reporting, the adoption of a common framework and universal language is crucial to optimize service deliver and improve functional outcomes worldwide.

In an effort to standardize the description of health, functional abilities, and disabilities of individuals in a context of a health condition, the World Health Organization (WHO) created the International Classification of Functioning, Disability and Health (ICF) as a reference family classification [20]. The ICF emphasizes the functional component of health. Conceptually, functioning represents what a person *can do or is able to do every day*, a more concrete and practical notion of “health”. (Video S1. Introduction to the ICF model *animation*—https://youtu.be/4kA-cRFn5Lo Supplementary Materials).

The ICF structures health and health-related domains into a hierarchy starting with components, then chapters, followed by *categories*. An ICF category is represented by an alphanumeric code denoting one of the components of the ICF: body functions [b], body structures [s], activities and participation [d], and environmental factors [e]. The component index letters are followed by a numeric code starting with the chapter number adding one digit, (e.g., **e1** Products and technology), followed by a second level category code adding two digits (e.g., e1**15** Products and technology for personal use), and third and fourth level code by adding one digit, respectively (e.g., e315**2** Products and technology for play and e3152**1** Adapted products and technology for play). In 2007, a Child and Youth version of the ICF, the ICF-CY [21], was published specifically to capture functioning in developing individuals by adding and expanding on the descriptions of categories provided in the ICF [20] in the following we use “ICF” to refer to both manuals).

**Figure 1:**
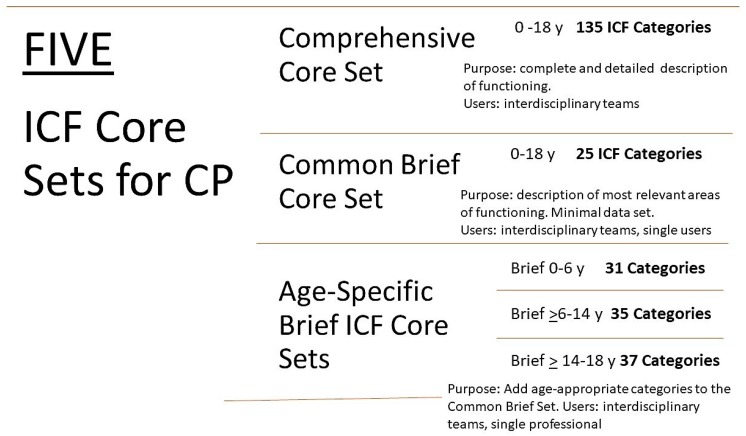
International Classification of Functioning, Disability, and Health (ICF) Core Sets for children and youth with Cerebral Palsy.

To facilitate the application of the ICF in day-to-day practice, shorter and more user-friendly ICF-based tools, so called ICF Core Sets, have been developed, which represent shortlists of ICF categories that cover the most relevant areas of functioning and disability in a specific condition. To date, ICF Core Sets have been developed for three childhood onset-disabilities: CP [22], autism spectrum disorder (ASD) [23], and attention-deficit-hyperactivity disorder (ADHD) [24]. These ICF Core Set for common childhood onset-disabilities capture unique functional areas for each condition [25]. The ICF Core Sets for CP offer service providers and stakeholders an age-appropriate framework to explore functioning and disability for assessment, treatment, evaluation, and policy purposes in a global context [26]. As shown in Figure 1, there are five ICF Core Sets for CP, a comprehensive and a common core sets covering ages 0 to 18 years, and three age-specific core sets capturing relevant functional developmental information. (Figure 1) User instructions and detailed content of each ICF Core Set for CP [22] is provided in Table S1.

Since the publication of the ICF Core Sets for CP in 2015, knowledge translation initiatives have been conducted to facilitate the adoption of these core sets worldwide. An open access educational website—entitled ICF educational e-tool [27]—was created illustrating how to apply the core sets in clinical practice using case scenarios. The website incorporates an abilities-oriented approach to assessment and treatment focused on the child’s and family’s preferences and the fundamental role of the environment facilitating functioning and disability, most importantly, using a positive language. The ICF educational e-tool is currently available in English and Portuguese. In addition, numerous educational activities were organized, such as e-courses in English (available open access at the ICFEducation.org website) and Spanish, and workshops in Poland, China, Argentina, Paraguay, United States (US), Mexico, Taiwan, Guatemala, Brazil, and Colombia. Furthermore, the ICF Core Sets for CP have been cultural validated in Poland, India, Pakistan, Taiwan, and Iran [28,29,30,31].

Of note, the ICF Core Sets for CP are dynamic, and it is expected that after their global application the content of the Core Sets will be revised and will evolve over time. Therefore, it is important to learn from ongoing initiates applying the ICF Core Sets for CP. As such, the overall purpose of this paper is to provide concrete examples of the application of the ICF Core Sets for children and youth with CP in four countries—Russia, Poland, Malawi, and Brazil—in different rehabilitation settings. The specific objective is as follows: to describe global initiatives applying the ICF Core Sets for CP, with a focus on contributions to clinical practice and challenges in their implementation.

## 2. Materials and Methods

Four ongoing initiatives applying the ICF Core Sets for children and youth with CP were included, as follows:

### 2.1. Brazil—Group Level Application—Congenital ZIKA Virus

- *Context:* The outbreak of Zika in Brazil in 2015–16 has had harmful medical, financial, and social consequences for many children and their families and significantly increased the statistics of Brazilian children with disabilities being followed up at rehabilitation centers. Many families face additional challenges in their daily lives due to poverty as well as structural issues related to the provision of health care and social security. From 2000 to 2014, 2464 live births with microcephaly were recorded in Brazil, with an annual average of 164 cases. In 2015, the number of cases increased nine times in relation to this average, totaling 1608 cases, which led the Ministry of Health to declare a public health emergency [32]. Seventy-one percent of live births with microcephaly were from the Northeast region of the country [33].
- *Purpose:* To describe the profile of functioning and disability of children with microcephaly following congenital Zika virus infection using an ICF-based tool.
- *Participants and Procedures*: The convenience sample consisted of 34 children with ZIKV-associated microcephaly in two states of northeastern Brazil, treated at four rehabilitation services in Paraíba and Rio Grande do Norte states. The Brazilian Portuguese version of the Brief Common ICF Core Set for CP was used. Each ICF category was assigned a qualifier, which ranged from 0 to 4 (no problem, mild problem, moderate problem, severe problem, complete problem).
  *Training raters:* each participating center held ICF and ICF Core Set training workshops using the ICF educational e-tool (http://learn.phsa.ca/shhc/icf/story_html5.html), as developed by Schiariti et al., 2015 [27]. Training was conducted by E.L. and H.F. (co-authors). The goal of the workshops was to consolidate the ICF theoretical and practical concepts, and to ensure the high inter-rater reliability of the qualifiers in each ICF Core Set category. Overall, a 16-h educational module was completed by health professionals that were involved in the study.
  *Translating clinical information into ICF qualifiers in Brazil:* The qualifiers to obtain the functional profile were generated through sensitive, reliable and validated instruments in Brazil [34], such as: Pediatric Evaluation of Disability Inventory (PEDI); Gross Motor Function Measure (GMFM—88); Visual Analog Scale (EVA); Infant Sleep Questionnaire (ISQ); Modified Ashworth Scale and Goniometry. Most of these measures are recommended in the ICF-based toolbox of measures aligning with the content of the CP core sets [35]. Scores were converted into ICF qualifiers either using clinical judgment—teamwork—or from visual response cards. For the categories that did not have available tools, a specific questionnaire was applied to parents or caregivers, whose responses were converted through the visual response cards into ICF qualifiers. The socio-demographic characteristics, cephalic perimeter, and other clinical data were collected through medical records, physical exams, imaging reports, and interviews with the children and their respective parents. Using the ICF-based documentation form, the ICF Core Set for CP was populated to create the profile of functioning of the sample [36]. Licensed physiotherapists in each center, who were familiar with each standardized test and completed the ICF training, were in charge of administering the tests and also translating the information into the ICF qualifiers. They were supervised by professor E.L. (co-author).
*Some examples on how clinical information was translated into the ICF qualifiers is provided below:*
  ○ The category s110 (structure of brain) was determined while considering the results of imaging exams (Nuclear Magnetic Resonance, Computed Tomography, or Trans fontanel Ultrasonography). For example, Computed Tomography demonstrating multiple calcifications at the cortical-white matter, predominating in temporal lobes, the qualifier 3 was assigned.
  ○ The category d710 (Basic interpersonal interactions) was captured by the PEDI, using the Social function area, items F and G, interactive social game, and interaction with friends, respectively. When the answer was 0, the qualifier 4 was assigned.
  ○ The category e120 (Products and technology for personal indoor and outdoor mobility and transportation) was assessed by a self-developed question: “Does the child need assistive devices to help in locomotion? How much does this help or hinder the child’s functioning?” To translate this information into the ICF language, firstly, the caregiver stated if the assistive device was considered to be a facilitator or a barrier. Secondly, the caregiver’s perspective on how much this environmental factor influenced the child’s functioning was captured while using the study visual response card. The caregiver’s response was mapped into the ICF qualifier (facilitator or barrier) [34].

### 2.2. Russia—System Level Application

- *Context*: CP represents a leading cause of childhood physical disability in Russia with an estimated prevalence of 2–4 cases per 1000 live births [37] (the multiple health and social services for CP available in the country, there is no systematic or standardized care pathway for this population. To obtain national and internationally comparable data in the assessment of CP, and to evaluate the effectiveness of rehabilitation efforts for children with CP, standardized tools are required).
- *Purpose:* To adopt the ICF Core Set in Russia as a guiding tool to design rehabilitation and habilitation programs for children with CP.
- *Participants and Procedures:* Participants were recruited from the Medical Social Expertise Service in St. Petersburg and Voronezh region. Overall rehabilitation programmes for 142 children were developed. Participants ages ranged from 1.5 to 18 years old, 34.4% of them were children under six years old, 36.6% were from seven to 13 and 28.8% were from 14 to 18.
- A rehabilitation algorithm was designed for CP, as follows:
  ○ Identification of key concerns impacting daily functioning.
  ○ Application of the Comprehensive and Common Brief ICF Core Sets for CP to be used as guiding framework for assessments and evaluations.
  ○ Building a profile of functioning for each child, and subsequently identifying rehabilitation tasks, including key ICF categories to target as goals for intervention.
  ○ Selection of rehabilitation instruments and therapeutic interventions aligning with the rehabilitation plan.

An interdisciplinary team of specialists, which included a neurologist, pediatrician, orthopedist, ophthalmologist, otolaryngologist, psychologist, psychiatrist, and social worker, conducted the examinations. Each of the specialists evaluated the ICF categories according to their specializations. It should be noted that all actions between them were coordinated to ensure interprofessional collaboration. Examples of tools, examinations, and questionnaires that were used to operationalize the Brief Common ICF Core Set for CP in Russia are provided in Table S2. 

### 2.3. Poland—Rehabilitation Center Application

- *Context*: The *Step by Step Association for help of disabled children in Zamość* (Poland) was established in 1990 and is a non-governmental organization uniting parents, caregivers, and friends of children, adolescents and disabled adults. (complete information here http://www.spdn.pl/) The center was founded by Dr Maria Krol, physician and mother of a young man with CP. Her caring and passionate work has provided a supportive and exceptional educational and rehabilitation center for children and youth with disabilities and their families in Zamość. The city of Zamość is in southeastern Poland; with a population of approximately 70,000 people. The historical centre of Zamość was added to the UNESCO World Heritage List in 1992. The *Step by Step Association for help of disabled children* is unique in the region of south-eastern Poland and leads professional activities in the field of integrated rehabilitation, education and social support of children, adolescents, and disabled adults with early brain damage. It includes comprehensive rehabilitation, education, and care for approximately 2000 people (150 children in daily care system—primarily the Integrated Rehabilitation System, 100 disabled people are covered by daily occupational therapy, and 1,800 people are in outpatient care). Services in the region are provided in eight facilities (Zamość centre, Biłgoraj centre, Occupation Therapy Workshops in Zamość and Biłgoraj, Centres for Social Activation in Zamość and Białobrzegi, Day Care Centre for Adults in Białobrzegi and Therapy and Recreation Park in Bondyrz). The Zamość centre leads day care, pre-school education, primary, secondary, and high school classes, which prepare adolescents for vocational training and employment.
- *Purpose*: To incorporate the ICF Core Sets for CP to guide the assessment process of children and youth with CP.
- *Participants and Procedures:* Children and youth with neurodevelopmental disabilities, including CP, spina bifida, and acquired brain injuries, attend the *Step by Step Association for help of disabled children.* The Zamość centre serves children from infancy to young adults (until the age of 25 years). Children are referred to the centre by physicians, psychologists, and other specialists, and they can also come directly without referral. Since the publication of the ICF Core Sets for children and youth with CP in 2015, the *Step by Step* has validated the tools using caregivers’ and clients’ perspectives. Following the ICF Core Sets user instructions, the team uses the ICF Core Sets as a condition-specific framework to select the most appropriate valid and reliable assessment tools. The child or youth’ perspectives about his/her functional abilities and expectations are incorporated as goals for interventions.

### 2.4. Malawi—Community-Based Rehabilitation Application

- *Context:* Malawi is a low-income country and it sits among the poorest countries in the world. Despite Malawian’s efforts to improve health care in the last years, there still exists barriers to accessing health system. The World Report on Disability from WHO shows that besides 83.4% of people needs health services, but only 61% really receive it. The same challenge is seen in the provision of rehabilitation services: 59.6% people with disabilities need rehabilitation services, but only 23.8% of them receive it. There are no data from CP prevalence in Malawi, but some studies in African countries shows a prevalence of 2–10 per 1000 children in community-based samples. Moreover, there is no national data regarding the impact of CP on everyday functioning and disability in pediatric populations.
- *Purpose:* To inspect the feasibility of the implementation of the ICF Core Sets for CP in a Malawian pediatric population diagnosed with CP.
- *Participants and Procedures:* Participants consisted of children between 0 to 18 years, recruited from the CBR program of an NGO operating in Malawi. Recruitment included 18 children and youth with CP. All participants were recruited in a rural area, where secondary and tertiary health care services are only in nearby cities, about two or three hours away A health care professional, familiar with the use of the ICF guided the assessments (C.V.). A local team collaborated in the project (D.S., M.M.). Information was gathered during clinical interviews and examinations. Additional information that was provided by the children and/or caregivers was also collected and linked to the ICF. The content validity of the ICF Core Sets was evaluated while using the frequency and percentage of subjects who had a strength/problem in each category.

## 3. Results

The four ongoing initiatives included in this study applied the ICF Core Sets for CP. Based on the reporting of data and feedback from professionals, the most user-friendly and feasible core set was the Common Brief ICF Core Set. A description of how the teams summarized functional data while using the ICF Core Sets is provided below.

### 3.1. Brazil—Group Level

Table 1 shows the participants’ characteristics. The majority of the children were girls, average age was 21 months, presented spastic bilateral CP, Gross Motor Function Classification System (GMFCS) [38,39] levels IV–V, and a head circumference with a z-score between 0.92 and −5.51. While using the Common Brief ICF core set, a profile of functioning was built for the entire study sample.

**Figure 2:**
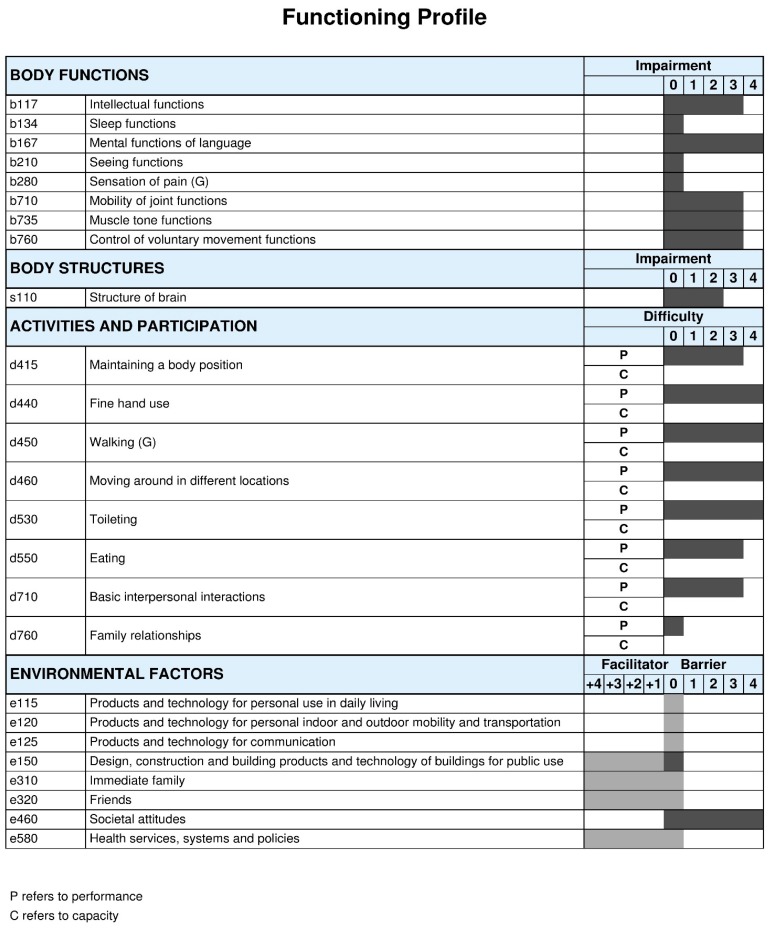
Functioning profile of the Brazilian children with cerebral palsy (CP). The ICF qualifiers use to create the profile of functioning of this study sample represent the qualifiers that have the highest percentage within each ICF category. This profile of functioning was built while using the ICF-based documentation form on this web page https://icf-core-sets.org/es/page0.php courtesy ICF Research Branch.

As shown in Figure 2, the profile of functioning provides a more detailed representation of the abilities and challenges of the children that are affected with Zika virus in the Northeast region of Brazil. The functioning profile revealed complete disability in most of the body function categories and the activity and participation areas were highly impacted, mainly in mobility-related categories. Regarding environmental factors, most of the participants reported a complete facilitator for immediate family, friends, and health services, systems, and policies, as well as a complete barrier to societal attitudes. Detailed results were recently published [34] Complete distribution of ICF qualifiers for the entire Brazilian sample is provided in Tables S3 and S4. 

ICF Qualifiers in body functions, body structures and activities and participation: 0 = no problem; 1 = mild problem; 2 = moderate problem; 3 = severe problem; and, 4 = complete problem. 20 ICF Qualifiers in environmental factors: 0 = no barrier/facilitator; +1 = mild facilitator; +2 = moderate facilitator; +3 = substantial facilitator; +4 = complete facilitator; 1 = mild barrier; 2 = moderate barrier; 3 = substantial barrier; and, 4 = complete barrier. For all components, ICF qualifiers 8 = not specified and 9 = not applicable. The component personal factors (pf) does not have ICF categories assigned, therefore it is recommended to add themes representing personal factors to complement the profile of functioning.

### 3.2. Russia–-System Level Applilcation

Table 1 shows the participants’ characteristics. The sample included 142 children with CP and represented all GMFCS levels. The majority were school-aged children and male. For this study, the profile of functioning and disability was built using the Common Brief ICF Core Set.

Table 2 shows the distribution of ICF qualifiers for children GMFCS levels I to III (*n* = 74) by ICF categories. Children presented several areas of strengths (no problem). However, as expected for CP, areas of body functions that are related to neuromuscular domains (b7) were the most affected, as well as areas of activities and participation related to mobility (d4), self-care (d5), and interpersonal relationships (d7). On the contrary, contextual factors were identified mainly as facilitators. 

ICF Qualifiers in body functions. body structures and activities and participation: 0 = no problem; 1 = mild problem; 2 = moderate problem; 3 = severe problem; and, 4 = complete problem. ICF Qualifiers in environmental factors: 0 = no barrier/facilitator; +1 = mild facilitator; +2 = moderate facilitator; +3 = substantial facilitator; +4 = complete facilitator; 1 = mild barrier; 2 = moderate barrier; 3 = substantial barrier; 4 = complete barrier. The component personal factors (pf) does not have ICF categories assigned, therefore it is recommended to add themes representing personal factors to complement the profile of functioning. P = performance. 

Table 3 shows the distribution of ICF qualifiers for children in GMFCS levels IV to V (*n* = 68). As expected, areas in body functions differed from children classified as GMFCS levels I to III, showing a more severe compromise of control of voluntary movements, muscle tone, and intellectual functions. In the area of activities and participation, children with CP with GMFCS levels IV to V, showed moderate limitations in all domains, expect for immediate family relationships, illustrating the importance of a supportive family environment.

ICF Qualifiers in body functions, body structures and activities and participation: 0 = no problem; 1 = mild problem; 2 = moderate problem; 3 = severe problem; and, 4 = complete problem [20]. ICF Qualifiers in environmental factors: 0 = no barrier/facilitator; +1 = mild facilitator; +2 = moderate facilitator; +3 = substantial facilitator; +4 = complete facilitator; 1 = mild barrier; 2 = moderate barrier; 3 = substantial barrier; and, 4 = complete barrier. The component personal factors (pf) does not have ICF categories assigned, therefore it is recommended to add themes representing personal factors to complement the profile of functioning.

**Figure 3:**
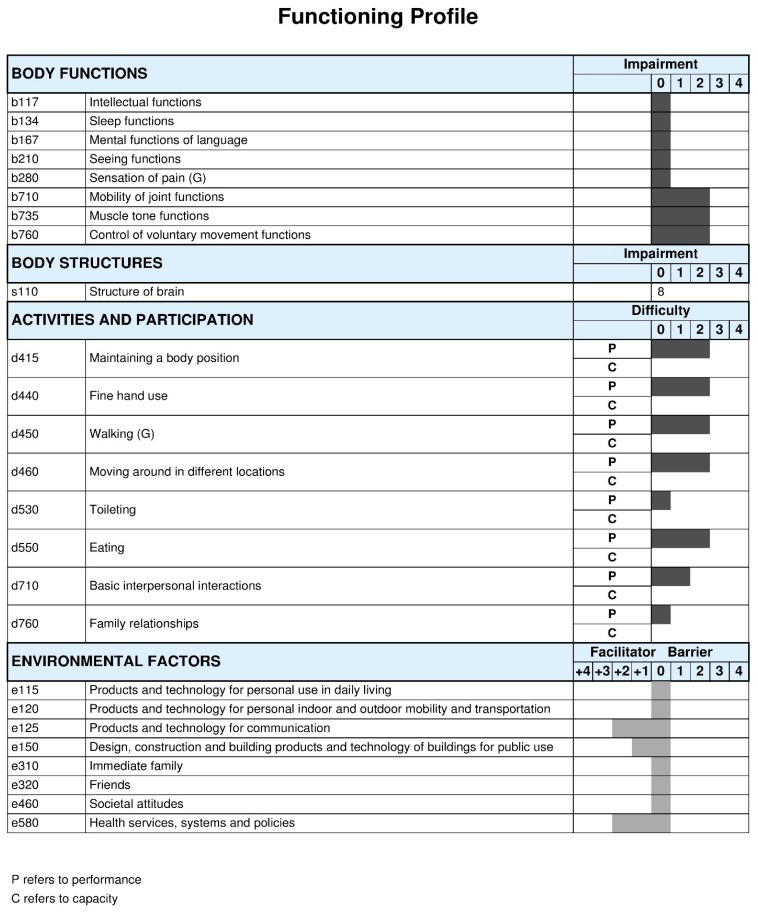
Functioning profile of the Russian children, GMFCS levels I to III. The ICF qualifiers use to create the profile of functioning of this study sample represent the qualifiers that have the highest percentage within each ICF category.

**Figure 4:**
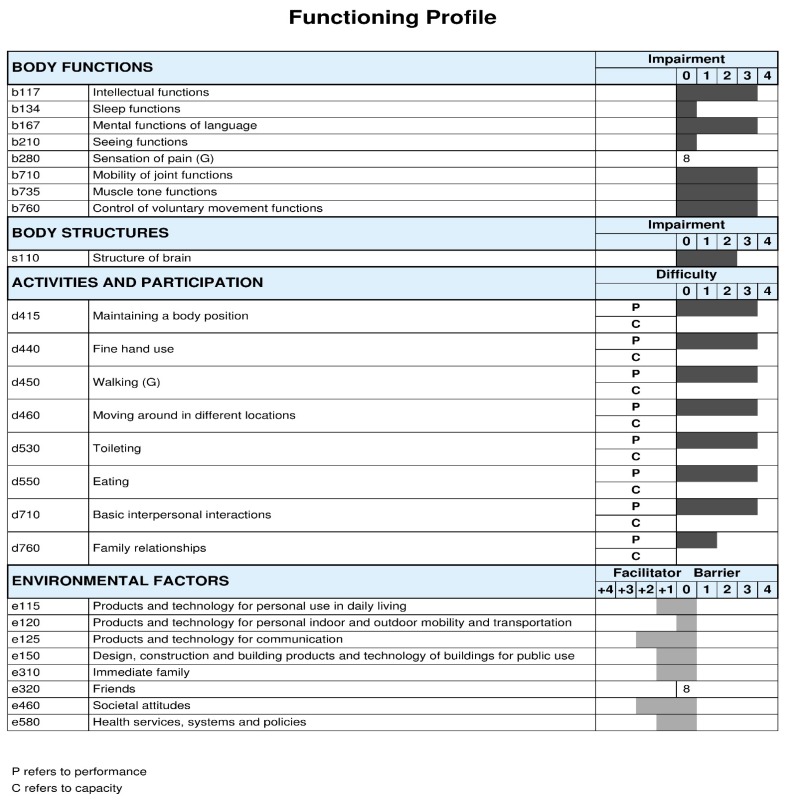
Functioning profile of the Russian children, GMFCS levels IV to V. The ICF qualifiers use to create the profile of functioning of this study sample represent the qualifiers that have the highest percentage within each ICF category.

Furthermore, Figure 3 and Figure 4 summarize the profile of functioning of Russian children with CP GMFCS levels I to III and GMFCS levels IV to V, respectively. Again, the functional profiles of this Russian population vary accordingly to GMFCS levels, with more children presenting impairments in body functions and more activity limitations and participation restrictions with higher GMFCS levels. However, the role of contextual factors influencing functioning, in general, have been reported as being a facilitator, in particular use of assistive technologies, services, and policies, as well as societal attitudes.

ICF Qualifiers in body functions, body structures and activities and participation: 0 = no problem; 1 = mild problem; 2 = moderate problem; 3 = severe problem; and, 4 = complete problem [20]. ICF Qualifiers in environmental factors: 0 = no barrier/facilitator; +1 = mild facilitator; +2 = moderate facilitator; +3 = substantial facilitator; +4 = complete facilitator; 1 = mild barrier; 2 = moderate barrier; 3 = substantial barrier; and, 4 = complete barrier. The component personal factors (pf) does not have ICF categories assigned, therefore it is recommended to add themes representing personal factors to complement the profile of functioning.

ICF Qualifiers in body functions, body structures and activities and participation: 0 = no problem; 1 = mild problem; 2 = moderate problem; 3 = severe problem; and, 4 = complete problem. ICF Qualifiers in environmental factors: 0 = no barrier/facilitator; +1 = mild facilitator; +2 = moderate facilitator; +3 = substantial facilitator; +4 = complete facilitator; 1 = mild barrier; 2 = moderate barrier; 3 = substantial barrier; and, 4 = complete barrier. The component personal factors (pf) does not have ICF categories assigned, therefore it is recommended to add themes representing personal factors to complement the profile of functioning.

### 3.3. Poland—Rehabilitation Center Application

#### 3.3.1. Service Delivery Model and the ICF in Zamość

**Figure 5:**
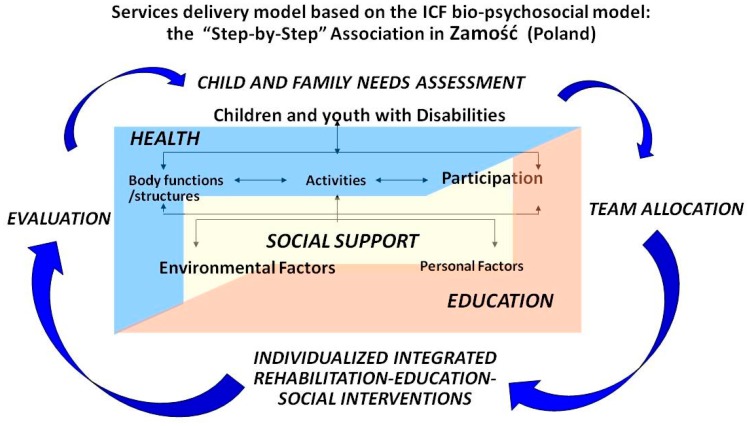
Service delivery model based on the ICF bio-psychosocial model at the Step by Step Association for help of disabled children in Zamość (Poland).

Figure 5 illustrates the integrated multidimensional service delivery model, based on the ICF model, adopted by the Step by Step Association for help of disabled children in Zamość. As shown in Figure 5, the ICF framework including its components of body functions and structures, activities and participation, and environmental and personal factors are a central part of planning services for each child or youth with neurodevelopmental disabilities. The health, education, and social support sectors are represented by a truly collaborative care team. The ICF components, specifically functional areas of participation and contextual factors are addressed across sectors, including relevant perspectives from professionals from the health, education, and social support sectors. Of note, as promoted by the ICF, the Step by Step Association promotes child and family-centered care by involving families’ priorities, preferences, and expectations when setting goals for interventions. Individualized Integrated Programmes are the basis for rehabilitation, education and social interventions in Zamość. Finally, the parents and caregivers are active members of the rehabilitation team delivering some interventions. Hence, parents and caregivers’ participation is a fundamental part of therapeutic interventions.

#### 3.3.2. Service Delivery Model and the ICF Core Sets for CP in Zamość

The ICF Core Sets are embedder in the delivery of service process in Zamość, which follows four steps—needs assessments, team allocation, individualized interventions, and evaluation—this cycle is completed every six months to ensure that appropriate services are offered to every child or youth. It is expected that the participation of the child or youth in every step of the decision-making process of this habilitation/rehabilitation cycle increase as the child grows. The Common Brief Core Set was the most frequently used as a guiding framework. 

### 3.4. Malawi—Community-Based Rehabilitation Application

In Malawi, the Common Brief ICF Core Set facilitated a holistic functional approach, guiding the assessment process, and promoting collaborative work. Clinical information was mapped into the Common Brief ICF Core Set. It was shown that not all of the areas included in this brief core sets were captured in day to day practice. (Table S5—Supplementary Materials). In body functions, as expected areas related to chapter b7, Neuromusculoskeletal areas were identified as being a major impairment. Some areas that were covered by the ICF Core Set, such as b280 pain and b134 sleep pattern, were not addressed by tools currently in used in Malawi. In activities and participation, several areas were identified as a strength by most of the group, including fine motor, eating, interpersonal relationships and moving (*n* = 12, 10, 10, and 9, respectively). It was impossible to assess familiar relationships (*n* = 18), as there were no standardized tools available. Several environmental factors were identified as facilities, such as family, friends, and products, and technology for mobility. On the contrary, some environmental factors remained a major barrier, including the lack of access to communication assistive devices, products and technology for daily living, and architectural design of public buildings.

## 4. Discussion

This is the first study describing practical applications of the ICF Core Sets for children and youth with CP worldwide. We have illustrated the multiple applications of these ICF-based tools in different pediatric contexts in Brazil, Russia, Poland, and Malawi. The study shows that the ICF Core Sets probe to be a useful tool to operationalize the biopsychosocial model—capturing social and medical information in a systematic way. From these initiatives, the most popular and feasible core set to summarize results and build profiles of functioning is the Common Brief ICF Core Set, this is probably explained for its short content (25 ICF categories) and the target age group (from 0 to 18 years of age). Due to its characteristics, the Common Brief Core Set has been proposed as the guiding tool to collect *minimal functional data* for CP from infancy to transition to adulthood.

In Brazil, the Common Brief ICF Core Set guided the description of the profile of functioning and disability of a prevalent health condition, like congenital ZIKA virus, which remains a public health concern due to its severe impact on the central nervous system and developmental trajectory of affected infants. Pioneer work from Professor Longo and her colleagues has shown how to standardize the collection of functional information of this population to complement diagnostic information, and most importantly, to plan resources, training, and interventions for this population for the years to come.

In Russia, the Comprehensive and Common Brief ICF Core Sets for CP have been used as a framework to standardize the assessment, evaluation, and treatment of children and youth with CP at a national level. This novel initiative is an example of the application of the ICF Core Set at a meso level (systems and services) and it shows how the core sets can be applied to delineate national care pathways for CP.

In Poland, the ICF has been used since its publication in 2001, showing their commitment to apply a holistic and integrative approach to childhood disability. The Step by Step Association was one of the first centers that organized educational sessions to facilitate a rapid adoption of the ICF Core Sets for CP. Zamosc has become a national reference for a comprehensive and successful approach to rehabilitation, which is mainly because the Step by Step Association features family-centered care, community inclusion, vocational rehabilitation, and a truly daily inter-professional collaboration approach.

In Malawi, the ICF Core Sets for CP were tested as a feasible tool to guide community-based rehabilitation for children and youth with CP. This small initiative was very valuable when considering the setting and resources that professionals had at their disposition. Nevertheless, initiatives like this provide useful information to improve community resources and expand community-based rehabilitation programs in middle and low-income countries.

### 4.1. Contributions and Challenges of Using the ICF Core Sets for CP

All in all, there were positive feedback and many contributions of adopting the ICF Core Sets for CP in clinical practice. In Russia, the implementation had a rapid adoption due to the availability of electronic health records, which facilitated the sharing of information between all members of the team, monitoring of rehabilitation stages—including both initial assessments and follow-up evaluations. Colleagues in Russia reported that the main contribution was the standardization of relevant areas of functioning and data collection, regardless of professionals’ experience or discipline. On the other hand, the main challenge of implemented the ICF Core Sets was the initial identification of Russian measures and examination tools to operationalize the content of the ICF Core Sets [40]. Local activities were carried out to reach consensus among experts and stakeholders regarding which set of tools were recommended for national use. Once this task was completed, the teams were able to proceed without major challenges.

In Brazil, the main advantage of using the ICF Core Sets was the adoption of a common language and comprehensive approach to describe the functional abilities and limitations of children with congenital Zika virus syndrome, beyond the anatomical description of the impact of ZIKV on the central nervous system. Another benefit was having a practical communication tool to share with families, building together the profile of functioning of each child, as families actively participated in the reporting and rating of categories. The main challenge was the lack of familiarity with the ICF model and ICF language, which required many educational sessions and the training of personnel as well as parents and caregivers. In addition, another challenge that the team encountered was the selection of appropriate measures validated in Brazil to assess the ICF categories of the core sets. 

In Poland and Malawi, the guiding framework of the ICF helped the teams to structure their daily approach and to systematically incorporate the families’ and the children’s perspectives on functioning. When considering the multiple needs of these populations and scarce resources, for example, lack of electronic records, reduce access to diagnostic testing, lack of qualified local trained professionals, and lack of research assistants and/or graduate students to collect and report data, some areas of the core sets were not reported. Overall, the main challenges were related to the lack of cultural accessible standardized tools, time allocation, and manpower to address all of the areas proposed by the ICF Core Sets.

Knowing that the ICF Core Sets for CP highlight *what* to measure but *not how* to measure relevant areas of functioning and disability, it is not surprising that teams encountered this as a major challenge of their implementation. To that end, the developers of the ICF Core Sets recently published a recommended toolbox of measure to address how to measure the components of the ICF Core Sets [35]. Further work is needed to complement this toolbox with culturally valid and reliable measures in different languages.

Finally, the implementation of the ICF Core Sets for CP standardized assessments, evaluations, and service provision in these global initiatives, promoting children with CP received effective and coordinated care. Despite the benefits of standardization, the ICF Core Sets for CP also allowed for the personalization of care, by adding as many ICF categories—as needed—to the proposed core set if they were considered relevant for a child. Thus, using the ICF Core Sets professionals can standardized and individualized care, features that are shared with other childhood-onset disabilities ICF Core Sets, such as ASD and ADHD [25], and other newly developed data collection standardization initiatives for CP [41,42].

### 4.2. ICF Core Sets for CP and Impact on Outcomes, Clinical Pracice, and Policy-Making

It is expected that the adoption of the ICF Core Sets for CP will drive positive change. Firstly, shifting needs assessment from a purely diagnostic and/or impairment-based approach to a comprehensive functional approach. For example, the profile of functioning—showing participation restrictions—could be used as an indicator of need for supports and services. As such, clinicians could use functional information to assess eligibility criteria for accessing services. Secondly, improvement in everyday functioning could be used as a desirable outcome for programme evaluation and policy-making. Lastly, the profile of functioning could be used to compare functional data to inform social policies and promote equality in providing services for the CP population. For example, children with same level of functioning receiving same environmental support across regions and levels of care.

### 4.3. Future Directions

Many of the initiates described in this paper are still ongoing, it is expected that “lessons learned” will be shared in the future, consequently revisions and practical recommendations will be formulated to enhance global adoption.

Our colleagues in Brazil are moving forward, they recognize the need to guide effective interventions and the identification of a set of *outcome measures* capturing the needs of this peculiar population. Thus, to elaborate a specific set of outcomes for the monitoring of children with congenital Zika virus infection and to standardize the results that are measured and reported in all Zika studies, a project in collaboration with the University of Liverpool (project leader Dr. Melissa Gladstone) has been launched—called the “Zika COS project: A study to develop a Core Outcome Set for children affected by congenital Zika virus”. A questionnaire based on the ICF Core Sets for CP [14,22] is currently being used in focus groups with parents of children that are affected by congenital Zika virus. (Table S6) In addition, an e-survey collecting health professionals’ perspectives regarding relevant outcomes is also underway. Interestingly, every family that participates in the study receives an early intervention package (handmade toys and instructions) that is based on the ICF model (Figure S1).

## 5. Conclusions

This study shows the multiple applications of the ICF Core Sets for CP in different contexts around the world, highlighting the benefits of standardizing assessments and evaluations in this population. Our findings probe that the implementation of the ICF Core Sets for CP is feasible. The ICF Core Sets for CP are helpful tools for clinicians and families, as theoretical frameworks, communication tools, functional classification tools, and as a method of comparing health and functional information. Reporting of these experiences has the potential to motivate other colleagues into adopting a bio-psychosocial model in a systematic way in their practices; learn from their challenges; and, prepare ahead to facilitate their implementation, ultimately improving global service provision for CP. 

## Figures and Tables

**Table 1:** Participants; characteristics by country.

	BRAZIL N = 34	RUSSIA N = 142	MALAWI N = 18
AGE (years)	Mean = 1.8 ± SD 6	Mean = 5.4 ± SD 3.74	8.6 ± SD 5.0
Gender	Male 47%	Male 65%	Male 44%
GMFCS level (%)	IV (32.4%), V (67.6%)	I (4%) II (16%) III (32%) IV (21%) V (26%)	III (27.8%), IV (27.8%), V (44.4%)
Type of CP (%)	Spastic Bilateral (76.5%)	Spastic Bilateral (72%)	NA
*Comorbidities*			
Intellectual disabilities	97.1%	29%	NA
Learning or behavioural disabilities	NA	33%	NA
Vision difficulties	67.6%	4%	NA
Epilepsy	NA	12%	11.1%
Caregiver characteristics	**Monthly Income** Up to 300 US dollars = 67.7% 300 to 600 US dollars = 35.3%	NA	NA
	**Schooling** Complete elementary education = 17.6% Incomplete elementary education = 14.7% Complete secondary education = 47.1% Others = 20.6%	Incomplete elementary education = 14.3 % (including home education = 0.9%) Incomplete secondary education = 50.0 % (including home education = 22.3%) Others = 12.5%	NA

NA; not available.

**Table 2:** Functioning profile of the Russian children with CP, Gross Motor Function Classification System (GMFCS) levels I to III (*n* = 74).

Brief ICF Core Set for CP		ICF Qualifiers, % Distribution among Children GMFCS Levels I to III									
		0	1	2	3	4	8	9			
b117		66.67	22.22	7.41	3.70	0.00	0.00	0.00			
b134		96.43	3.57	0.00	0.00	0.00	0.00	0.00			
b167		39.29	21.43	35.71	3.57	0.00	0.00	0.00			
b210		75.00	25.00	0.00	0.00	0.00	0.00	0.00			
b280		53.57	0.00	0.00	0.00	0.00	46.43	0.00			
b710		0.00	10.71	71.43	17.86	0.00	0.00	0.00			
b735		0.00	0.00	71.43	28.57	0.00	0.00	0.00			
b760		0.00	0.00	74.07	22.22	3.70	0.00	0.00			
		0	1	2	3	4	8	9			
s110	degree	10.71	14.29	21.43	0.00	0.00	53.57	0.00			
	nature of the change	11.11	14.81	22.22	0.00	0.00	51.85	0.00			
	location	11.11	14.81	22.22	0.00	0.00	51.85	0.00			
		0	1	2	3	4	8	9			
d415	Р	0.00	0.00	75.00	21.43	3.57	0.00	0.00			
d440	Р	7.14	0.00	64.29	25.00	3.57	0.00	0.00			
d450	Р	0.00	0.00	65.38	30.77	3.85	0.00	0.00			
d460	Р	0.00	0.00	70.37	25.93	3.70	0.00	0.00			
d530	Р	67.86	25.00	0.00	7.14	0.00	0.00	0.00			
d550	Р	10.71	7.14	67.86	10.71	3.57	0.00	0.00			
d710	Р	29.63	48.15	18.52	3.70	0.00	0.00	0.00			
d760	Р	71.43	25.00	3.57	0.00	0.00	0.00	0.00			
	4	3	2	1	0	1	2	3	4	8	9
e115	0	0.00	32.14	14.29	53.57	0.00	0.00	0.00	0.00	0.00	0.00
e120	0	0	0	0	0	0	0	0	0	0	100
e125	0	0.00	62.50	12.50	25.00	0.00	0.00	0.00	0.00	0.00	0.00
e150	0	0.00	3.57	35.71	50.00	0.00	0.00	0.00	0.00	10.71	0.00
e310	10.71	0.00	7.14	14.29	60.71	3.57	0.00	0.00	0.00	3.57	0.00
e320	0	4.00	0.00	8.00	72.00	4.00	0.00	0.00	0.00	12.00	0.00
e460	0	0.00	0.00	0.00	87.50	12.50	0.00	0.00	0.00	0.00	0.00
e580	0	0.00	37.50	25.00	37.50	0.00	0.00	0.00	0.00	0.00	0.00

**Table 3:** Functioning profile of the Russian children with CP, GMFCS levels IV to V (*n* = 68).

Brief ICF Core Set for CP		ICF Qualifiers, % Distribution among Children GMFCS Levels IV to V									
%		0	1	2	3	4	8	9
b117		3.03	9.09	21.21	51.52	15.15	0.00	0.00
b134		75.76	15.15	9.09	0.00	0.00	0.00	0.00
b167		9.09	9.09	15.15	51.52	15.15	0.00	0.00
b210		63.64	30.30	0.00	0.00	6.06	0.00	0.00
b280		39.39	0.00	0.00	0.00	0.00	60.61	0.00
b710		3,03	0.00	33.33	48.48	15.15	0.00	0.00
b735		0.00	0.00	6.06	66.67	27.27	0.00	0.00
b760		0.00	0.00	0.00	69.70	30.30	0.00	0.00
		0	1	2	3	4	8	9
s110	degree	3.03	6.06	33.33	24.24	3.03	30.30	0.00
	nature of the change	3.03	6.06	33.33	24.24	3.03	30.30	0.00
	location	3.03	6.06	33.33	24.24	3.03	30.30	0.00
		0	1	2	3	4	8	9
d415	Р	0.00	0.00	0.00	68.75	31.25	0.00	0.00
d440	Р	0.00	0.00	0.00	68.75	31.25	0.00	0.00
d450	Р	0.00	0.00	3.23	67.74	29.03	0.00	0.00
d460	Р	0.00	0.00	0.00	68.75	31.25	0.00	0.00
d530	Р	6.25	6.25	0.00	53.13	9.38	25.00	0.00
d550	Р	0.00	6.25	6.25	59.38	28.13	0.00	0.00
d710	Р	0.00	9.38	15.63	65.63	9.38	0.00	0.00
d760	Р	25.00	50.00	9.38	12.50	3.13	0.00	0.00			
	4	3	2	1	0	1	2	3	4	8	9
e115	0	6.25	31.25	59.38	3.13	0.00	0.00	0.00	0.00	0.00	0.00
		
e120	0	0	2.82	0	0	0	0	0	0	0	96.48
		
e125	0	28.57	28.57	0.00	0.00	0.00	0.00	0.00	0.00	28.57	14.29
		
e150	0	0.00	0.00	77.42	9.68	0.00	0.00	0.00	0.00	12.90	0.00
e310	3,03	0.00	21.21	51.52	18.18	0.00	0.00	0.00	0.00	6.06	0.00
		
e320	0	0.00	0.00	37.50	9.38	0.00	0.00	0.00	0.00	46.88	6.25
		
e460	0	25.00	0.00	25.00	25.00	25.00	0.00	0.00	0.00	0.00	0.00
		
e580	0	20.00	0.00	60.00	20.00	0.00	0.00	0.00	0.00	0.00	0.00
		

**P**; performance.

## Supplementary Materials

The following are available online at http://www.mdpi.com/1660-4601/15/9/1899/s1, Video S1: Introduction to ICF model and application in Cerebral Palsy-animation https://youtu.be/4kA-cRFn5Lo, Table S1: ICF Core Sets for children and youth with CP complete list with user instructions, Table S2: Examples of tools, examinations, and questionnaires used to apply the Brief Common ICF Core Set for CP in Russia, Table S3: Distribution of ICF qualifiers entire Brazilian sample with congenital ZIKA virus (Body functions and structures, and activities and participation), Table S4: Distribution of ICF qualifiers entire Brazilian sample with congenital ZIKA virus (environmental factors), Table S5: Profile of functioning of Malawi children with CP, *n* = 18; Table S6: Interview guideline for ongoing ZIKA COS study—based on ICF Core Sets for CP, Figure S1: Illustration of early intervention package for participants of ZIKA COS study—based on the ICF model—Brazilian initiative.
