# Supplementary material for: Implementation of the International Classification of Functioning, Disability, and Health (ICF) Core Sets for Children and Youth with Cerebral Palsy: Global Initiatives Promoting Optimal Functioning
Source: Int J Environ Res Public Health. 2018 Sep 1;15(9):1899. doi: 10.3390/ijerph15091899 (PMC6163506; doi:10.3390/ijerph15091899)
Supplement: Supplementary file 1 [file ijerph-15-01899-s001.pdf]

International Journal of
Environmental Research and Public Health

MDPI

Supplementary Material – VIDEO S1

Introduction to the ICF model in pediatrics, link to open-access video:

https://youtu.be/4kA-cRFn5Lo

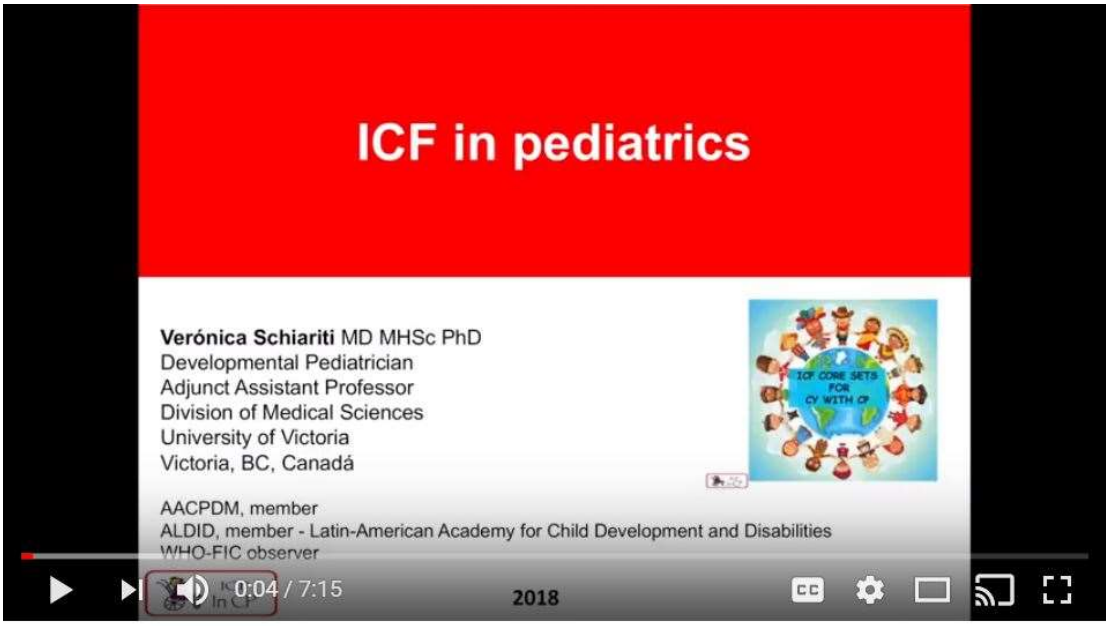

This video briefly introduces the ICF model, its dimensions and components. A practical case scenario illustrates how to navigate the model in a regular clinical situation. Finally, it highlights the importance of adopting the ICF model in pediatrics to ensure the application of the conventions of people with disabilities and the rights of children.

Table S1. ICF Core Sets for children and youth with CP complete list with user instructions [22].

ICF CORE SETS FOR CHILDREN AND YOUTH WITH CEREBRAL PALSY - USER INSTRUCTIONS

The purpose of the ICF Core Sets for children and youth (CY) with cerebral palsy (CP) is to describe the present child or youth's level of functioning, along with facilitators and barriers that influence functioning. The ICF Core Sets for CY with CP can be applied in clinical, research, teaching and administration. Emphasis is on the child or youth's performance of everyday activities at home, school and in the community.

There are five ICF Core Sets for CY with CP: the Comprehensive ICF Core Set, the Common Brief ICF Core Set, and the three Age-Specific Brief ICF Core Sets (0 to 6 years, 6≥ to <14, and 14≥ to 18 years of age). User instructions are provided to assist in determining how to select and use the Core Sets.

Step I – Selection of Type of ICF Core Set

The selection of the appropriate type of ICF Core Set – Comprehensive, Common Brief, or Age-Specific Brief – depends on the user's intended purpose and the amount of information he or she requires to characterize the level of functioning.

- *Comprehensive ICF Core Set for CY with CP:* Used for a complete and detailed description of functioning in CY with CP aged 0 to 18 years. Due to the level of detail, this Core Set is useful in interdisciplinary

assessments for which an exhaustive assessment can be distributed among team members from different professions.

- *Common Brief ICF Core Set for CY with CP:* Used to describe the most common areas of functioning in CY aged 0 to 18 years with CP. The Common Brief Core Set can be employed in regular clinical encounters where only a brief assessment is necessary, and in clinical and epidemiological research. This Core Set can be used by a single professional or an inter-disciplinary team. A key characteristic of the Common Brief Core Set is that it allows the description of the level of functioning of CY with CP over time, from birth to transition to adulthood. Therefore, this Core Set can be considered as a minimum for any "minimal data set" which describes CY with CP across their lifespan. Use of this Core Set by administrators, health planners, and CP registries would be expected.
- *Age-Specific Brief ICF Core Set for CY with CP:* Each of the age-specific sets includes the ICF categories of the Common Brief ICF Core Set in addition to ICF categories unique to each age-group: 0 to <6, 6≥ to <14 and 14≥ to 18 years of age. Each age-specific ICF Core Set describes the most common areas of functioning in CY with CP for the associated age-group. The Age-Specific ICF Core Sets can be applied in clinical or research context where additional detail is desirable in caring for or describing a particular age-group of CY with CP. These ICF Core Sets can be used by a single professional or an interdisciplinary team.

Step II- Description of Level of Functioning

Once the appropriate ICF Core Set is selected, the ICF categories that are contained in the ICF Core Set are used as a reference to guide users through the assessment process. Each ICF category has a definition with inclusion and exclusion criteria. (pediatric ICF manual) Professionals may use interviews (medical history), clinical examination, clinical tools, self or proxy-reported questionnaires, and/or technical investigations to collect information related to each ICF category. The selection of questionnaires and clinical tools is dependent on how well the content of the tools align with the ICF categories that are included in the Core Set. (For examples on how to select a tool using the ICF as a reference please see Schiariti et al.). **Note:** work is under way to identify a pool of measures that best represent the content of the five ICF Core Sets for CY with CP.

Step III- Rating the Degree of Functioning

Professionals may choose to summarize the information collected during the assessment process and create a functional profile of the child or youth with CP (e.g. Figure 2). For this purpose, ICF qualifiers must be assigned to each of the core set's ICF categories. The ICF qualifiers represent a general ordinal scale ranging from 0=no problem to 4=complete problem (pediatric ICF manual). The ICF qualifiers describe the degree of impairment, limitation or restriction in each category within the component *Body Structures, Body Functions, and Activity and Participation*. Additionally, within the component *Environmental Factors* the ICF qualifiers describe barriers or facilitators, ranging from 0=no barrier/ facilitator to 4 complete barrier/facilitator, denoting facilitators with a "+". The assignment of ICF qualifiers is made using clinical judgement and they should only be used as descriptors of the degree of functioning. They do not represent an assessment tool. We recommend clinicians working in multidisciplinary teams to discuss the assignment of ICF qualifiers before using the rating scale in clinical practice. We also recommend that users establish intrarater reliability before using the ICF qualifiers for research purposes.

Finally, as the component *Personal Factors* does not have ICF categories assigned, we also recommend adding to the functional profile the essential personal characteristics of the child or youth with CP that would have a positive or negative impact on *Body Structures, Body Functions, Activity and Participation,* and *Environmental Factors*. *Personal Factors,* such as preferences, positive attitudes,

and other attributes can influence adherence to interventions; thus, they should be routinely addressed and considered by professionals.

It is note-worthy that the ICF Core Sets for CY with CP allow the description of both abilities and limitations in everyday functioning, along with facilitators and barriers that influence functioning. We believe that the inclusion of positive aspects (abilities, strengths and facilitators) when describing functioning is a significant contribution of the ICF Core Sets for CY with CP, encouraging professionals and families to share an optimistic approach to the functioning of CY with CP.

The five ICF Core Sets for CY with CP are provided below:

BRIEF COMMON ICF CORE SET FOR CHILDREN & YOUTH WITH CEREBRAL PALSY
From 0 to 18 years
N=25 ICF Categories

**BODY STRUCTURES (n=1)**<br>= anatomical parts of the body such as organs, limbs and their components	
**s110**	**Structure of brain**

**BODY FUNCTIONS (n=8)**<br>= physiological functions of body systems (including psychological functions)	
**b117**	**Intellectual functions**
	**General mental functions, required to understand and constructively integrate the various mental functions, including all cognitive functions and their development over the life span.**<br>*Inclusions: functions of intellectual growth; intellectual retardation, mental retardation, dementia*<br>*Exclusions: memory functions (b144); thought functions (b160); basic cognitive functions (b163); higher-level cognitive functions (b164)*
**b134**	**Sleep functions**
	**General mental functions of periodic, reversible and selective physical and mental disengagement from one's immediate environment accompanied by characteristic physiological changes.**<br>*Inclusions: functions of amount of sleeping, and onset, maintenance and quality of sleep; functions involving the sleep cycle, such as in insomnia, hypersomnia and narcolepsy*<br>*Exclusions: consciousness functions (b110); energy and drive functions (b130); attention functions (b140); psychomotor functions (b147)*
**b167**	**Mental functions of language**
	**Specific mental functions of recognizing and using signs, symbols and other components of a language.**<br>*Inclusions: functions of reception and decryption of spoken, written or other forms of language such as sign language; functions of expression of spoken, written or other forms of language; integrative language functions, spoken and written, such as involved in receptive, expressive, Broca's, Wernicke's and conduction aphasia*<br>*Exclusions: attention functions (b140); memory functions (b144); perceptual functions (b156); thought functions (b160); higher-level cognitive functions (b164); calculation functions (b172); mental functions of complex movements (b176); Chapter 2 Sensory Functions and Pain; Chapter 3 Voice and Speech Functions*
**b210**	**Seeing functions**
	**Sensory functions relating to sensing the presence of light and sensing the form, size, shape and colour of the visual stimuli.**<br>*Inclusions: visual acuity functions; visual field functions; quality of vision; functions of sensing light and colour, visual acuity of distant and near vision, monocular and binocular vision; visual picture quality; impairments such as myopia, hypermetropia, astigmatism, hemianopia, colour-blindness, tunnel vision, central and peripheral scotoma, diplopia, night blindness and impaired adaptability to light*<br>*Exclusion: perceptual functions (b156)*
**b280**	**Sensation of pain**
	**Sensation of unpleasant feeling indicating potential or actual damage to some body structure.**<br>*Inclusions: sensations of generalized or localized pain in one or more body part, pain in a dermatome, stabbing pain, burning pain, dull pain, aching pain; impairments such as myalgia, analgesia and hyperalgesia*
**b710**	**Mobility of joint functions**
	**Functions of the range and ease of movement of a joint.**<br>*Inclusions: functions of mobility of single or several joints, vertebral, shoulder, elbow, wrist, hip, knee, ankle, small joints of hands and feet; mobility of joints generalized; impairments such as in hypermobility of joints, frozen joints, frozen shoulder, arthritis Exclusions: stability of joint functions (b715); control of voluntary movement functions (b760)*

**b735**	**Muscle tone functions**
	**Functions related to the tension present in the resting muscles and the resistance offered when trying to move the muscles passively.**<br>*Inclusions: functions associated with the tension of isolated muscles and muscle groups,muscles of one limb, one side of the body and the lower half of the body, muscles of all limbs, muscles of the trunk, and all muscles of the body; impairments such as hypotonia, hypertonia and muscle spasticity, myotonia and paramyotonia*<br>*Exclusions: muscle power functions (b730); muscle endurance functions (b740)*
**b760**	**Control of voluntary movement functions**
	Functions associated with control over and coordination of voluntary movements.<br>*Inclusions: functions of control of simple voluntary movements and of complex voluntary movements, coordination of voluntary movements, supportive functions of arm or leg, right left motor coordination, eye hand coordination, eye foot coordination; impairments such as control and coordination problems, e.g. clumsiness and dysdiadochokinesia*<br>*Exclusions: muscle power functions (b730); involuntary movement functions (b765); gait pattern functions (b770)*

**ACTIVITIES AND PARTICIPATION (n=8)**<br>= execution of a task or action by an individual and involvement in a life situation	
**d415**	**Maintaining a body position**
	**Staying in the same body position as required, such as remaining seated or remaining standing for work or school.**<br>*Inclusions: maintaining a lying, squatting, kneeling, sitting and standing position*
**d440**	**Fine hand use**
	**Performing the coordinated actions of handling objects, picking up, manipulating and releasing them using one's hand, fingers and thumb, such as required to lift coins off a table or turn a dial or knob.**<br>*Inclusions: picking up, grasping, manipulating and releasing*
**d450**	**Walking**
	**Moving along a surface on foot, step by step, so that one foot is always on the ground, such as when strolling, sauntering, walking forwards, backwards or sideways.**<br>*Inclusions: walking short or long distances; walking on different surfaces; walking around obstacles Exclusions: transferring oneself (d420); moving around (d455)*<br>*Exclusions: transferring oneself (d420); moving around (d455)*
**d460**	**Moving around in different locations**
	**Walking and moving around in various places and situations, such as walking between rooms in a house, within a building, or down the street of a town..**<br>*Inclusions: moving around within the home, crawling or climbing within the home; walking or moving within buildings other than the home, and outside the home an dother buildings*
**d530**	**Toileting**
	**Indicating the need for, planning and carrying out the elimination of human waste (menstruation, urination and defecation), and cleaning oneself afterwards.**<br>*Inclusions: regulating urination, defecation and menstrual care*<br>*Exclusions: washing oneself (d510); caring for body parts (d520)*
**d550**	**Eating**
	**Indicating need for, and carrying out the coordinated tasks and actions of eating food that has been served, bringing it to the mouth and consuming it in culturally acceptable ways, cutting or breaking food into pieces, opening bottles and cans, using eating implements, having meals, feasting or dining.**<br>*Exclusion: drinking (d560)*
**d710**	**Basic interpersonal interactions**
	**Interacting with people in a contextually and socially appropriate manner, such as by showing consideration and esteem when appropriate, or responding to the feelings of others.**<br>*Inclusions: showing respect, warmth, appreciation, and tolerance in relationships; responding to criticism and social cues in relationships; and using appropriate physical contact in relationships*
**d760**	**Family relationships**
	**Creating and maintaining kinship relationships, such as with members of the nuclear family, extended family, foster and adopted family and step-relationships, more distant relationships such as second cousins or legal guardians.**<br>*Inclusions: parent-child and child-parent relationships, sibling and extended family relationships*

ENVIRONMENTAL FACTORS (n=8)<br>= make up the physical, social and attitudinal environment in which people live and conduct their lives	
**e115**	**Products and technology for personal use in daily living**
	**Equipment, products and technologies used by people in daily activities, including those adapted or specially designed, located in, on or near the person using them.**<br>*Inclusions: general and assistive products and technology for personal use*<br>*Exclusions: products and technology for personal indoor and outdoor mobility and transportation (e120); products and technology for communication (e125)*
**e120**	**Products and technology for personal indoor and outdoor mobility and transportation**
	**Equipment, products and technologies used by people in activities of moving inside and outside buildings, including those adapted or specially designed, located in, on or near the person using them.**<br>*Inclusions: general and assistive products and technology for personal indoor and outdoor mobility and transportation*
**e125**	**Products and technology for communication**
	**Equipment, products and technologies used by people in activities of sending and receiving information, including those adapted or specially designed, located in, on or near the person using them.**<br>*Inclusions: general and assistive products and technology for communication*
**e150**	**Design, construction and building products and technology of buildings for public use**
	**Products and technology that constitute an individual's indoor and outdoor human-made environment that is planned, designed and constructed for public use, including those adapted or specially designed.**<br>*Inclusions: design, construction and building products and technology of entrances and exits, facilities and routing*
**e310**	**Immediate family**
	**Individuals related by birth, marriage or other relationship recognized by the culture as immediate family, such as spouses, partners, parents, siblings, children, foster parents, adoptive parents and grandparents.**<br>*Exclusions: extended family (e315); personal care providers and personal assistants (e340)*
**e320**	**Friends**
	**Individuals who are close and ongoing participants in relationships characterized by trust and mutual support.**
**e460**	**Societal attitudes**
	**General or specific opinions and beliefs generally held by people of a culture, society, subcultural or other social group about other individuals or about other social, political and economic issues that influence group or individual behaviour and actions.**
**e580**	**Health services, systems and policies**
	**Services, systems and policies for preventing and treating health problems, providing medical rehabilitation and promoting a healthy lifestyle.**<br>*Exclusion: general social support services, systems and policies (e575)*

Note: because of the hierarchical order of the classification, including a second level category automatically includes the third and fourth level categories listed underneath the second level category. Complete list of categories can be found at the ICF Children and Youth browser: http://apps.who.int/classifications/icfbrowser/Default.aspx

BRIEF ICF CORE SET FOR CHILDREN & YOUTH WITH CEREBRAL PALSY,
From 0 to <6 years of age
N= 31 ICF Categories
*** Green-marked items from the Common ICF Core Set**

BODY STRUCTURES (n=1)<br>= anatomical parts of the body such as organs, limbs and their components	
**s110**	**Structure of brain**

BODY FUNCTIONS (n=9)<br>= physiological functions of body systems (including psychological functions)	
**b117**	**Intellectual functions**
	**General mental functions, required to understand and constructively integrate the various mental functions, including all cognitive functions and their development over the life span.**<br>*Inclusions: functions of intellectual growth; intellectual retardation, mental retardation, dementia*<br>*Exclusions: memory functions (b144); thought functions (b160); basic cognitive functions (b163); higher-level cognitive functions (b164)*

**BODY FUNCTIONS (n=9)**<br>= physiological functions of body systems (including psychological functions)	
**b134**	**Sleep functions**
	**General mental functions of periodic, reversible and selective physical and mental disengagement from one's immediate environment accompanied by characteristic physiological changes.**<br>*Inclusions: functions of amount of sleeping, and onset, maintenance and quality of sleep; functions involving the sleep cycle, such as in insomnia, hypersomnia and narcolepsy*<br>*Exclusions: consciousness functions (b110); energy and drive functions (b130); attention functions (b140); psychomotor functions (b147)*
**b167**	**Mental functions of language**
	**Specific mental functions of recognizing and using signs, symbols and other components of a language.**<br>*Inclusions: functions of reception and decryption of spoken, written or other forms of language such as sign language; functions of expression of spoken, written or other forms of language; integrative language functions, spoken and written, such as involved in receptive, expressive, Broca's, Wernicke's and conduction aphasia*<br>*Exclusions: attention functions (b140); memory functions (b144); perceptual functions (b156); thought functions (b160); higher-level cognitive functions (b164); calculation functions (b172); mental functions of complex movements (b176); Chapter 2 Sensory Functions and Pain; Chapter 3 Voice and Speech Functions*
**b210**	**Seeing functions**
	**Sensory functions relating to sensing the presence of light and sensing the form, size, shape and colour of the visual stimuli.**<br>*Inclusions: visual acuity functions; visual field functions; quality of vision; functions of sensing light and colour, visual acuity of distant and near vision, monocular and binocular vision; visual picture quality; impairments such as myopia, hypermetropia, astigmatism, hemianopia, colour-blindness, tunnel vision, central and peripheral scotoma, diplopia, night blindness and impaired adaptability to light*<br>*Exclusion: perceptual functions (b156)*
**b230**	**Hearing functions**
	**Sensory functions relating to sensing the presence of sounds and discriminating the location, pitch, loudness and quality of sounds.**<br>*Inclusions: functions of hearing, auditory discrimination, localization of sound source, lateralization of sound, speech discrimination; impairments such as deafness, hearing impairment and hearing loss*<br>*Exclusions: perceptual functions (b156) and mental functions of language (b167)*
**b280**	**Sensation of pain**
	**Sensation of unpleasant feeling indicating potential or actual damage to some body structure.**<br>*Inclusions: sensations of generalized or localized pain in one or more body part, pain in a dermatome, stabbing pain, burning pain, dull pain, aching pain; impairments such as myalgia, analgesia and hyperalgesia*
**b710**	**Mobility of joint functions**
	**Functions of the range and ease of movement of a joint.**<br>*Inclusions: functions of mobility of single or several joints, vertebral, shoulder, elbow, wrist, hip, knee, ankle, small joints of hands and feet; mobility of joints generalized; impairments such as in hypermobility of joints, frozen joints, frozen shoulder, arthritis*<br>*Exclusions: stability of joint functions (b715); control of voluntary movement functions (b760)*
**b735**	**Muscle tone functions**
	**Functions related to the tension present in the resting muscles and the resistance offered when trying to move the muscles passively.**<br>*Inclusions: functions associated with the tension of isolated muscles and muscle groups, muscles of one limb, one side of the body and the lower half of the body, muscles of all limbs, muscles of the trunk, and all muscles of the body; impairments such as hypotonia, hypertonia and muscle spasticity, myotonia and paramyotonia*<br>*Exclusions: muscle power functions (b730); muscle endurance functions (b740)*
**b760**	**Control of voluntary movement functions**
	Functions associated with control over and coordination of voluntary movements.<br>*Inclusions: functions of control of simple voluntary movements and of complex voluntary movements, coordination of voluntary movements, supportive functions of arm or leg, right left motor coordination, eye hand coordination, eye foot coordination; impairments such as control and coordination problems, e.g. clumsiness and dysdiadochokinesia*<br>*Exclusions: muscle power functions (b730); involuntary movement functions (b765);*

**BODY FUNCTIONS (n=9)**<br>= physiological functions of body systems (including psychological functions)	
	gait pattern functions (b770)

**ACTIVITIES AND PARTICIPATION (n=11)**<br>= execution of a task or action by an individual and involvement in a life situation	
d133	**Acquiring language**
	**Developing the competence to represent persons, objects, events and feelings through words, symbols, phrases and sentences.**<br>Exclusions: acquiring additional language (d134); communication (d310-d399)
d155	**Acquiring skills**
	**Developing basic and complex competencies in integrated sets of actions or tasks so as to initiate and follow through with the acquisition of a skill, such as manipulating tools or toys, or playing games.**<br>*Inclusions: acquiring basic and complex skills*<br>*Exclusions: learning to write (d145) and writing (d170), learning to play (d131)*
d415	**Maintaining a body position**
	**Staying in the same body position as required, such as remaining seated or remaining standing for work or school.**<br>*Inclusions: maintaining a lying, squatting, kneeling, sitting and standing position*
d440	**Fine hand use**
	**Performing the coordinated actions of handling objects, picking up, manipulating and releasing them using one's hand, fingers and thumb, such as required to lift coins off a table or turn a dial or knob.**<br>*Inclusions: picking up, grasping, manipulating and releasing*
d450	**Walking**
	**Moving along a surface on foot, step by step, so that one foot is always on the ground, such as when strolling, sauntering, walking forwards, backwards or sideways.**<br>*Inclusions: walking short or long distances; walking on different surfaces; walking around obstacles*<br>*Exclusions: transferring oneself (d420); moving around (d455)*
d460	**Moving around in different locations**
	**Walking and moving around in various places and situations, such as walking between rooms in a house, within a building, or down the street of a town..**<br>*Inclusions: moving around within the home, crawling or climbing within the home; walking or moving within buildings other than the home, and outside the home an dother buildings*
d530	**Toileting**
	**Indicating the need for, planning and carrying out the elimination of human waste (menstruation, urination and defecation), and cleaning oneself afterwards.**<br>*Inclusions: regulating urination, defecation and menstrual care*<br>*Exclusions: washing oneself (d510); caring for body parts (d520)*
d550	**Eating**
	**Indicating need for, and carrying out the coordinated tasks and actions of eating food that has been served, bringing it to the mouth and consuming it in culturally acceptable ways, cutting or breaking food into pieces, opening bottles and cans, using eating implements, having meals, feasting or dining.**<br>*Exclusion: drinking (d560)*
d710	**Basic interpersonal interactions**
	**Interacting with people in a contextually and socially appropriate manner, such as by showing consideration and esteem when appropriate, or responding to the feelings of others.**<br>*Inclusions: showing respect, warmth, appreciation, and tolerance in relationships; responding to criticism and social cues in relationships; and using appropriate physical contact in relationships*
d760	**Family relationships**

	**Creating and maintaining kinship relationships, such as with members of the nuclear family, extended family, foster and adopted family and step-relationships, more distant relationships such as second cousins or legal guardians.** *Inclusions: parent-child and child-parent relationships, sibling and extended family relationships*
d880	**Engagement in play**
	**Engagement in play** **Purposeful, sustained engagement in activities with objects, toys, materials or games, occupying oneself or with others.**

**ENVIRONMENTAL FACTORS (n=10)** = make up the physical, social and attitudinal environment in which people live and conduct their lives	
**e115**	**Products and technology for personal use in daily living**
	**Equipment, products and technologies used by people in daily activities, including those adapted or specially designed, located in, on or near the person using them.** *Inclusions: general and assistive products and technology for personal use* *Exclusions: products and technology for personal indoor and outdoor mobility and transportation (e120); products and technology for communication (e125)*
**e120**	**Products and technology for personal indoor and outdoor mobility and transportation**
	**Equipment, products and technologies used by people in activities of moving inside and outside buildings, including those adapted or specially designed, located in, on or near the person using them.** *Inclusions: general and assistive products and technology for personal indoor and outdoor mobility and transportation*
**e125**	**Products and technology for communication**
	**Equipment, products and technologies used by people in activities of sending and receiving information, including those adapted or specially designed, located in, on or near the person using them.** *Inclusions: general and assistive products and technology for communication*
**e150**	**Design, construction and building products and technology of buildings for public use**
	**Products and technology that constitute an individual's indoor and outdoor human-made environment that is planned, designed and constructed for public use, including those adapted or specially designed.** *Inclusions: design, construction and building products and technology of entrances and exits, facilities and routing*
**e310**	**Immediate family**
	**Individuals related by birth, marriage or other relationship recognized by the culture as immediate family, such as spouses, partners, parents, siblings, children, foster parents, adoptive parents and grandparents.** *Exclusions: extended family (e315); personal care providers and personal assistants (e340)*
**e320**	**Friends**
	**Individuals who are close and ongoing participants in relationships characterized by trust and mutual support.**
**e355**	**Health professionals**
	**All service providers working within the context of the health system, such as doctors, nurses, physiotherapists, occupational therapists, speech therapists, audiologists, orthotist-prosthetists, medical social workers.** *Exclusion: other professionals (e360)*
**e410**	**Individual attitudes of immediate family members**
	**General or specific opinions and beliefs of immediate family members about the person or about other matters (e.g. social, political and economic issues), that influence individual behaviour and actions.**
**e460**	**Societal attitudes**
	**General or specific opinions and beliefs generally held by people of a culture, society, subcultural or other social group about other individuals or about other social, political and economic issues that influence group or individual behaviour and actions.**
**e580**	**Health services, systems and policies**
	**Services, systems and policies for preventing and treating health problems, providing medical rehabilitation and promoting a healthy lifestyle.** *Exclusion: general social support services, systems and policies (e575)*

BRIEF ICF CORE SET FOR CHILDREN & YOUTH WITH CEREBRAL PALSY,
Aged 6 ≥ to <14 years of age
N=35 ICF Categories
*** Green-marked items from the Common ICF Core Set**

BODY STRUCTURES (n=1)<br>= anatomical parts of the body such as organs, limbs and their components	
**s110**	**Structure of brain**

BODY FUNCTIONS (n=10)<br>= physiological functions of body systems (including psychological functions)	
**b117**	**Intellectual functions**
	**General mental functions, required to understand and constructively integrate the various mental functions, including all cognitive functions and their development over the life span.**<br>*Inclusions: functions of intellectual growth; intellectual retardation, mental retardation, dementia*<br>*Exclusions: memory functions (b144); thought functions (b160); basic cognitive functions (b163); higher-level cognitive functions (b164)*
**b1301**	**Motivation**
**b134**	**Sleep functions**
	**General mental functions of periodic, reversible and selective physical and mental disengagement from one's immediate environment accompanied by characteristic physiological changes.**<br>*Inclusions: functions of amount of sleeping, and onset, maintenance and quality of sleep; functions involving the sleep cycle, such as in insomnia, hypersomnia and narcolepsy*<br>*Exclusions: consciousness functions (b110); energy and drive functions (b130); attention functions (b140); psychomotor functions (b147)*
**b140**	**Attention functions**
	**Specific mental functions of focusing on an external stimulus or internal experience for the required period of time.**<br>*Inclusions: functions of sustaining attention, shifting attention, dividing attention, sharing attention; concentration; distractibility*<br>*Exclusions: consciousness functions (b110); energy and drive functions (b130); sleep functions (b134); memory functions (b144); psychomotor functions (b147); perceptual functions (b156)*
**b167**	**Mental functions of language**
	**Specific mental functions of recognizing and using signs, symbols and other components of a language.**<br>*Inclusions: functions of reception and decryption of spoken, written or other forms of language such as sign language; functions of expression of spoken, written or other forms of language; integrative language functions, spoken and written, such as involved in receptive, expressive, Broca's, Wernicke's and conduction aphasia*<br>*Exclusions: attention functions (b140); memory functions (b144); perceptual functions (b156); thought functions (b160); higher-level cognitive functions (b164); calculation functions (b172); mental functions of complex movements (b176); Chapter 2 Sensory Functions and Pain; Chapter 3 Voice and Speech Functions*
**b210**	**Seeing functions**
	**Sensory functions relating to sensing the presence of light and sensing the form, size, shape and colour of the visual stimuli.**<br>*Inclusions: visual acuity functions; visual field functions; quality of vision; functions of sensing light and colour, visual acuity of distant and near vision, monocular and binocular vision; visual picture quality; impairments such as myopia, hypermetropia, astigmatism, hemianopia, colour-blindness, tunnel vision, central and peripheral scotoma, diplopia, night blindness and impaired adaptability to light*<br>*Exclusion: perceptual functions (b156)*
**b280**	**Sensation of pain**
	**Sensation of unpleasant feeling indicating potential or actual damage to some body structure.**<br>*Inclusions: sensations of generalized or localized pain in one or more body part, pain in a dermatome, stabbing pain, burning pain, dull pain, aching pain; impairments such as myalgia, analgesia and hyperalgesia*
**b710**	**Mobility of joint functions**
	**Functions of the range and ease of movement of a joint.**<br>*Inclusions: functions of mobility of single or several joints, vertebral, shoulder, elbow, wrist, hip, knee, ankle, small joints of hands and feet; mobility of joints generalized; impairments such as in hypermobility of joints, frozen joints, frozen shoulder, arthritis*<br>*Exclusions: stability of joint functions (b715); control of voluntary movement functions (b760)*
**b735**	**Muscle tone functions**
	**Functions related to the tension present in the resting muscles and the resistance offered when trying to move the muscles passively.**

**BODY FUNCTIONS (n=10)**<br>= physiological functions of body systems (including psychological functions)	
	Inclusions: functions associated with the tension of isolated muscles and muscle groups, muscles of one limb, one side of the body and the lower half of the body, muscles of all limbs, muscles of the trunk, and all muscles of the body; impairments such as hypotonia, hypertonia and muscle spasticity, myotonia and paramyotonia<br>Exclusions: muscle power functions (b730); muscle endurance functions (b740)
**b760**	**Control of voluntary movement functions**
	**Functions associated with control over and coordination of voluntary movements.**<br>Inclusions: functions of control of simple voluntary movements and of complex voluntary movements, coordination of voluntary movements, supportive functions of arm or leg, right left motor coordination, eye hand coordination, eye foot coordination; impairments such as control and coordination problems, e.g. clumsiness and dysdiadochokinesia<br>Exclusions: muscle power functions (b730); involuntary movement functions (b765);<br>gait pattern functions (b770)

**ACTIVITIES AND PARTICIPATION (n=13)**<br>= execution of a task or action by an individual and involvement in a life situation	
**d175**	**Solving problems**
	**Finding solutions to questions or situations by identifying and analysing issues, developing options and solutions, evaluating potential effects of solutions, and executing a chosen solution, such as in resolving a dispute between two people.**<br>*Inclusions: solving simple and complex problems*<br>*Exclusions: thinking (d163); making decisions (d177)*
**d230**	**Carrying out daily routine**
	**Carrying out simple or complex and coordinated actions in order to plan, manage and complete the requirements of day-to-day procedures or duties, such as budgeting time and making plans for separate activities throughout the day.**<br>*Inclusions: managing and completing the daily routine; managing one's own activity level*<br>*Exclusion: undertaking multiple tasks (d220)*
**d350**	**Conversation**
	**Starting, sustaining and ending an interchange of thoughts and ideas, carried out by means of spoken, written, sign or other forms of language, with one or more people one knows or who are strangers, in formal or casual settings.**<br>*Inclusions: starting, sustaining and ending a conversation; conversing with one or many people*
**d415**	**Maintaining a body position**
	**Staying in the same body position as required, such as remaining seated or remaining standing for work or school.**<br>*Inclusions: maintaining a lying, squatting, kneeling, sitting and standing position*
**d440**	**Fine hand use**
	**Performing the coordinated actions of handling objects, picking up, manipulating and releasing them using one's hand, fingers and thumb, such as required to lift coins off a table or turn a dial or knob.**<br>*Inclusions: picking up, grasping, manipulating and releasing*
**d450**	**Walking**
	**Moving along a surface on foot, step by step, so that one foot is always on the ground, such as when strolling, sauntering, walking forwards, backwards or sideways.**<br>*Inclusions: walking short or long distances; walking on different surfaces; walking around obstacles*<br>*Exclusions: transferring oneself (d420); moving around (d455)*
**d460**	**Moving around in different locations**
	**Walking and moving around in various places and situations, such as walking between rooms in a house, within a building, or down the street of a town..**<br>*Inclusions: moving around within the home, crawling or climbing within the home; walking or moving within buildings other than the home, and outside the home an dother buildings*
**d530**	**Toileting**

ACTIVITIES AND PARTICIPATION (n=13)<br>= execution of a task or action by an individual and involvement in a life situation	
	**Indicating the need for, planning and carrying out the elimination of human waste (menstruation, urination and defecation), and cleaning oneself afterwards.**<br>*Inclusions: regulating urination, defecation and menstrual care*<br>*Exclusions: washing oneself (d510); caring for body parts (d520)*
d550	**Eating**
	**Indicating need for, and carrying out the coordinated tasks and actions of eating food that has been served, bringing it to the mouth and consuming it in culturally acceptable ways, cutting or breaking food into pieces, opening bottles and cans, using eating implements, having meals, feasting or dining.**<br>*Exclusion: drinking (d560)*
d710	**Basic interpersonal interactions**
	**Interacting with people in a contextually and socially appropriate manner, such as by showing consideration and esteem when appropriate, or responding to the feelings of others.**<br>*Inclusions: showing respect, warmth, appreciation, and tolerance in relationships; responding to criticism and social cues in relationships; and using appropriate physical contact in relationships*
d760	**Family relationships**
	**Creating and maintaining kinship relationships, such as with members of the nuclear family, extended family, foster and adopted family and step-relationships, more distant relationships such as second cousins or legal guardians.**<br>*Inclusions: parent-child and child-parent relationships, sibling and extended family relationships*

ACTIVITIES AND PARTICIPATION (Continued)	
d820	**School education**
	**Gaining admission to school, engaging in all school-related responsibilities and privileges, and learning the course material, subjects and other curriculum requirements in a primary or secondary education programme, including attending school regularly, working cooperatively with other students, taking direction from teachers, organizing, studying and completing assigned tasks and projects, and advancing to other stages of education.**
d920	**Recreation and leisure**
	**Engaging in any form of play, recreational or leisure activity, such as informal or organized play and sports, programmes of physical fitness, relaxation, amusement or diversion, going to art galleries, museums, cinemas or theatres; engaging in crafts or hobbies, reading for enjoyment, playing musical instruments; sightseeing, tourism and travelling for pleasure.**<br>*Inclusions: games, sports, arts and culture, crafts, hobbies and socializing*<br>*Exclusions: riding animals for transportation (d480); remunerative and nonremunerative work (d850 and d855); engagement in play (d880); religion and spirituality (d930); political life and citizenship (d950)*

ENVIRONMENTAL FACTORS (n=11)<br>= make up the physical, social and attitudinal environment in which people live and conduct their lives	
e115	**Products and technology for personal use in daily living**
	**Equipment, products and technologies used by people in daily activities, including those adapted or specially designed, located in, on or near the person using them.**<br>*Inclusions: general and assistive products and technology for personal use*<br>*Exclusions: products and technology for personal indoor and outdoor mobility and transportation (e120); products and technology for communication (e125)*
e120	**Products and technology for personal indoor and outdoor mobility and transportation**

	Equipment, products and technologies used by people in activities of moving inside and outside buildings, including those adapted or specially designed, located in, on or near the person using them.<br>*Inclusions: general and assistive products and technology for personal indoor and outdoor mobility and transportation*
e125	Products and technology for communication
	Equipment, products and technologies used by people in activities of sending and receiving information, including those adapted or specially designed, located in, on or near the person using them.<br>*Inclusions: general and assistive products and technology for communication*
e130	Products and technology for education
	Equipment, products, processes, methods and technology used for acquisition of knowledge, expertise or skill, including those adapted or specially designed.<br>*Inclusion: general and assistive products and technology for education*
e140	Products and technology for culture, recreation and sport
	Equipment, products and technology used for the conduct and enhancement of cultural, recreational and sporting activities, including those adapted or specially designed.<br>*Inclusion: general and assistive products and technology for culture, recreation and sport*<br>*Exclusion: products and technology for play (e1152)*
e150	Design, construction and building products and technology of buildings for public use
	Products and technology that constitute an individual's indoor and outdoor human-made environment that is planned, designed and constructed for public use, including those adapted or specially designed.<br>*Inclusions: design, construction and building products and technology of entrances and exits, facilities and routing*
e310	Immediate family
	Individuals related by birth, marriage or other relationship recognized by the culture as immediate family, such as spouses, partners, parents, siblings, children, foster parents, adoptive parents and grandparents.<br>*Exclusions: extended family (e315); personal care providers and personal assistants (e340)*
e320	Friends
	Individuals who are close and ongoing participants in relationships characterized by trust and mutual support.
e460	Societal attitudes
	General or specific opinions and beliefs generally held by people of a culture, society, subcultural or other social group about other individuals or about other social, political and economic issues that influence group or individual behaviour and actions.
e580	Health services, systems and policies
	Services, systems and policies for preventing and treating health problems, providing medical rehabilitation and promoting a healthy lifestyle.<br>*Exclusion: general social support services, systems and policies (e575)*
e585	Education and training services, systems and policies
	Services, systems and policies for the acquisition, maintenance and improvement of knowledge, expertise and vocational or artistic skills. See UNESCO's International Standard Classification of Education (ISCED-1997).

BRIEF ICF CORE SET FOR CHILDREN & YOUTH WITH CEREBRAL PALSY,
Aged ≥14 to 18 years of age
N= 37 ICF Categories
* Green-marked items from the Common ICF Core Set

**BODY STRUCTURES (n=1)**<br>= anatomical parts of the body such as organs, limbs and their components	
s110	Structure of brain

**BODY FUNCTIONS (n=10)**<br>= physiological functions of body systems (including psychological functions)	
b117	Intellectual functions
	General mental functions, required to understand and constructively integrate the various mental functions, including all cognitive functions and their development over the life span.<br>*Inclusions: functions of intellectual growth; intellectual retardation, mental retardation, dementia*<br>*Exclusions: memory functions (b144); thought functions (b160); higher-level cognitive functions (b164)*
b1301	Motivation
b134	Sleep functions

	**General mental functions of periodic, reversible and selective physical and mental disengagement from one's immediate environment accompanied by characteristic physiological changes.**<br>*Inclusions: functions of amount of sleeping, and onset, maintenance and quality of sleep; functions involving the sleep cycle, such as in insomnia, hypersomnia and narcolepsy*<br>*Exclusions: consciousness functions (b110); energy and drive functions (b130); attention functions (b140); psychomotor functions (b147)*
**b164**	**Higher-level cognitive functions**
	**Specific mental functions especially dependent on the frontal lobes of the brain, including complex goal-directed behaviours such as decision-making, abstract thinking, planning and carrying out plans, mental flexibility, and deciding which behaviours are appropriate under what circumstances; often called executive functions.**<br>*Inclusions: functions of abstraction and organization of ideas; time management, insight and judgement; concept formation, categorization and cognitive flexibility*<br>*Exclusions: memory functions (b144); thought functions (b160); mental functions of language (b167); calculation functions (b172)*
**b167**	**Mental functions of language**
	**Specific mental functions of recognizing and using signs, symbols and other components of a language.**<br>*Inclusions: functions of reception and decryption of spoken, written or other forms of language such as sign language; functions of expression of spoken, written or other forms of language; integrative language functions, spoken and written, such as involved in receptive, expressive, Broca's, Wernicke's and conduction aphasia Exclusions: attention functions (b140); memory functions (b144); perceptual functions (b156); thought functions (b160); higher-level cognitive functions (b164); calculation functions (b172); mental functions of complex movements (b176); Chapter 2 Sensory Functions and Pain; Chapter 3 Voice and Speech Functions*
**b210**	**Seeing functions**
	**Sensory functions relating to sensing the presence of light and sensing the form, size, shape and colour of the visual stimuli.**<br>*Inclusions: visual acuity functions; visual field functions; quality of vision; functions of sensing light and colour, visual acuity of distant and near vision, monocular and binocular vision; visual picture quality; impairments such as myopia, hypermetropia, astigmatism, hemianopia, colour-blindness, tunnel vision, central and peripheral scotoma, diplopia, night blindness and impaired adaptability to light Exclusion: perceptual functions (b156)*
**b280**	**Sensation of pain**
	**Sensation of unpleasant feeling indicating potential or actual damage to some body structure.**<br>*Inclusions: sensations of generalized or localized pain in one or more body part, pain in a dermatome, stabbing pain, burning pain, dull pain, aching pain; impairments such as myalgia, analgesia and hyperalgesia*
**b710**	**Mobility of joint functions**
	**Functions of the range and ease of movement of a joint.**<br>*Inclusions: functions of mobility of single or several joints, vertebral, shoulder, elbow, wrist, hip, knee, ankle, small joints of hands and feet; mobility of joints generalized; impairments such as in hypermobility of joints, frozen joints, frozen shoulder, arthritis*<br>*Exclusions: stability of joint functions (b715); control of voluntary movement functions (b760)*
**b735**	**Muscle tone functions**
	**Functions related to the tension present in the resting muscles and the resistance offered when trying to move the muscles passively.**<br>*Inclusions: functions associated with the tension of isolated muscles and muscle groups, muscles of one limb, one side of the body and the lower half of the body, muscles of all limbs, muscles of the trunk, and all muscles of the body; impairments such as hypotonia, hypertonia and muscle spasticity, myotonia and paramyotonia*<br>*Exclusions: muscle power functions (b730); muscle endurance functions (b740)*
**BODY FUNCTIONS (n=10)**<br>= physiological functions of body systems (including psychological functions) (Continued)	
**b760**	**Control of voluntary movement functions**
	Functions associated with control over and coordination of voluntary movements.<br>*Inclusions: functions of control of simple voluntary movements and of complex voluntary movements, coordination of voluntary movements, supportive functions of arm or leg, right left motor coordination, eye hand coordination, eye foot coordination; impairments such as control and coordination problems, e.g. clumsiness and dysdiadochokinesia*<br>*Exclusions: muscle power functions (b730); involuntary movement functions (b765); gait pattern functions (b770)*

ACTIVITIES AND PARTICIPATION (n=15)<br>= execution of a task or action by an individual and involvement in a life situation	
d175	Solving problems
	**Finding solutions to questions or situations by identifying and analysing issues, developing options and solutions, evaluating potential effects of solutions, and executing a chosen solution, such as in resolving a dispute between two people.**<br>*Inclusions: solving simple and complex problems*<br>*Exclusions: thinking (d163); making decisions (d177)*
d250	Managing one’s own behaviour
	Carrying out simple or complex and coordinated actions in a consistent manner<br>in response to new situations, persons or experiences, such as being quiet in a<br>library.
d415	Maintaining a body position
	**Staying in the same body position as required, such as remaining seated or remaining standing for work or school.**<br>*Inclusions: maintaining a lying, squatting, kneeling, sitting and standing position*
d440	Fine hand use
	**Performing the coordinated actions of handling objects, picking up, manipulating and releasing them using one's hand, fingers and thumb, such as required to lift coins off a table or turn a dial or knob.**<br>*Inclusions: picking up, grasping, manipulating and releasing*
d450	Walking
	**Moving along a surface on foot, step by step, so that one foot is always on the ground, such as when strolling, sauntering, walking forwards, backwards or sideways.**<br>*Inclusions: walking short or long distances; walking on different surfaces; walking around obstacles Exclusions: transferring oneself (d420); moving around (d455)*
d460	Moving around in different locations
	**Walking and moving around in various places and situations, such as walking<br>between rooms in a house, within a building, or down the street of a town..**<br>*Inclusions: moving around within the home, crawling or climbing within the home; walking or moving within buildings other than the home, and outside the home an dother buildings*
d530	Toileting
	**Planning and carrying out the elimination of human waste (menstruation, urination and defecation), and cleaning oneself afterwards.**<br>*Inclusions: regulating urination, defecation and menstrual care*<br>*Exclusions: washing oneself (d510); caring for body parts (d520)*
d550	Eating
	**Carrying out the coordinated tasks and actions of eating food that has been served, bringing it to the mouth and consuming it in culturally acceptable ways, cutting or breaking food into pieces, opening bottles and cans, using eating implements, having meals, feasting or dining.**<br>*Exclusion: drinking (d560)*
d570	Looking after one’s health
	**Ensuring physical comfort, health and physical and mental well-being, such as by maintaining a balanced diet, and an appropriate level of physical activity, keeping warm or cool, avoiding harms to health, following safe sex practices, including using condoms, getting immunizations and regular physical examinations.**<br>*Inclusions: ensuring one’s physical comfort; managing diet and fitness; maintaining one’s health*
d710	Basic interpersonal interactions
	**Interacting with people in a contextually and socially appropriate manner, such as by showing consideration and esteem when appropriate, or responding to the feelings of others.**<br>*Inclusions: showing respect, warmth, appreciation, and tolerance in relationships; responding to criticism and social cues in relationships; and using appropriate physical contact in relationships*
d720	Complex interpersonal interactions

	**Maintaining and managing interactions with other people, in a contextually and socially appropriate manner, such as by regulating emotions and impulses, controlling verbal and physical aggression, acting independently in social interactions, and acting in accordance with social rules and conventions.** *Inclusions: forming and terminating relationships; regulating behaviours within interactions; interacting according to social rules; and maintaining social space*
ACTIVITIES AND PARTICIPATION (n=15) (Continued)	
d760	Family relationships
	**Creating and maintaining kinship relationships, such as with members of the nuclear family, extended family, foster and adopted family and step-relatioships, more distant relationships such as second cousins or legal guardians.** *Inclusions: parent-child and child-parent relationships, sibling and extended family relationships*
d820	School education
	**Gaining admission to school, engaging in all school-related responsibilities and privileges, and learning the course material, subjects and other curriculum requirements in a primary or secondary education programme, including attending school regularly, working cooperatively with other students, taking direction from teachers, organizing, studying and completing assigned tasks and projects, and advancing to other stages of education.**
d845	Acquiring, keeping and terminating a job
	**Seeking, finding and choosing employment, being hired and accepting employment, maintaining and advancing through a job, trade, occupation or profession, and leaving a job in an appropriate manner.** *Inclusions: seeking employment; preparing a resume or curriculum vitae; contacting employers and preparing interviews; maintaining a job; monitoring one's own work performance; giving notice; and terminating a job*
d920	Recreation and leisure
	**Engaging in any form of play, recreational or leisure activity, such as informal or organized play and sports, programmes of physical fitness, relaxation, amusement or diversion, going to art galleries, museums, cinemas or theatres; engaging in crafts or hobbies, reading for enjoyment, playing musical instruments; sightseeing, tourism and travelling for pleasure.** *Inclusions: play, sports, arts and culture, crafts, hobbies and socializing* *Exclusions: riding animals for transportation (d480); remunerative and non-remunerative work (d850 and d855); religion and spirituality (d930); political life and citizenship (d950)*

ENVIRONMENTAL FACTORS (n=11) = make up the physical, social and attitudinal environment in which people live and conduct their lives	
e115	**Products and technology for personal use in daily living**
	**Equipment, products and technologies used by people in daily activities, including those adapted or specially designed, located in, on or near the person using them.** *Inclusions: general and assistive products and technology for personal use*
e120	**Products and technology for personal indoor and outdoor mobility and transportation**
	**Equipment, products and technologies used by people in activities of moving inside and outside buildings, including those adapted or specially designed, located in, on or near the person using them.** *Inclusions: general and assistive products and technology for personal indoor and outdoor mobility and transportation*
e125	**Products and technology for communication**
	**Equipment, products and technologies used by people in activities of sending and receiving information, including those adapted or specially designed, located in, on or near the person using them.** *Inclusions: general and assistive products and technology for communication*
e150	**Design, construction and building products and technology of buildings for public use**
	**Products and technology that constitute an individual's indoor and outdoor human-made environment that is planned, designed and constructed for public use, including those adapted or specially designed.** *Inclusions: design, construction and building products and technology of entrances and exits, facilities and routing*
e310	**Immediate family**
	**Individuals related by birth, marriage or other relationship recognized by the culture as immediate family, such as spouses, partners, parents, siblings, children, foster parents, adoptive parents and grandparents.** *Exclusions: extended family (e315); personal care providers and personal assistants (e340)*

e320	Friends
	Individuals who are close and ongoing participants in relationships characterized by trust and mutual support.
e420	Individual attitude of friends
	General or specific opinions and beliefs of friends about the person or about other matters (e.g. social, political and economic issues) that influence individual behaviour and actions.
e460	Societal attitudes
	General or specific opinions and beliefs generally held by people of a culture, society, subcultural or other social group about other individuals or about other social, political and economic issues that influence group or individual behaviour and actions.
e540	Transportation services, systems and policies
	Services, systems and policies for enabling people or goods to move or be moved from one location to another.
e580	Health services, systems and policies
	Services, systems and policies for preventing and treating health problems, providing medical rehabilitation and promoting a healthy lifestyle.<br>*Exclusion: general social support services, systems and policies (e575)*
e585	Education and training services, systems and policies
	Services, systems and policies for the acquisition, maintenance and improvement of knowledge, expertise and vocational or artistic skills. See UNESCO's International Standard Classification of Education (ISCED-1997).

COMPREHENSIVE ICF CORE SET FOR CHILDREN & YOUTH WITH CEREBRAL PALSY FROM BIRTH TO 18 YEARS OF AGE

N= 135 ICF categories

* Green-marked items were identified for the Brief Common ICF Core Set

BODY STRUCTURES (N = 7)<br>= anatomical parts of the body such as organs, limbs and their components	
s110	Structure of brain
s320	Structure of mouth
s730	Structure of upper extremity
s750	Structure of lower extremity
s760	Structure of trunk
s7700	Bones
s7703	Extra-articular ligaments, fasciae, extramuscular aponeuroses, retinacula, septa, bursae, unspecified

BODY FUNCTIONS (N = 34)<br>= physiological functions of body systems (including psychological functions)	
b117	Intellectual functions
	General mental functions, required to understand and constructively integrate the various mental functions, including all cognitive functions and their development over the life span.<br>*Inclusions: functions of intellectual growth; intellectual retardation, mental retardation, dementia*<br>*Exclusions: memory functions (b144); thought functions (b160); basic cognitive functions (b163); higher-level cognitive functions (b164)*
b126	Temperament and personality functions
	General mental functions of constitutional disposition of the individual to react in a particular way to situations, including the set of mental characteristics that makes the individual distinct from others.<br>*Remark: The codes on Temperament and Personality functions can be related to the codes on expression of dispositions and intra-personal functions (b125). Users may use either or both. The taxonomic properties of these codes and their relationship need to be developed through research.*<br>*Inclusions: functions of extraversion, introversion, agreeableness, conscientiousness, psychic and emotional stability, and openness to experience; optimism; novelty seeking; confidence; trustworthiness*<br>*Exclusions: intellectual functions (b117); energy and drive functions (b130); psychomotor functions (b147); emotional functions (b152)*
b1301	Motivation
	Mental functions that produce the incentive to act; the conscious or unconscious driving force for action.
b134	Sleep functions

	**General mental functions of periodic, reversible and selective physical and mental disengagement from one's immediate environment accompanied by characteristic physiological changes.**<br>*Inclusions: functions of amount of sleeping, and onset, maintenance and quality of sleep; functions involving the sleep cycle, such as in insomnia, hypersomnia and narcolepsy*<br>*Exclusions: consciousness functions (b110); energy and drive functions (b130); attention functions (b140); psychomotor functions (b147)*
**b140**	**Attention functions**
	**Specific mental functions of focusing on an external stimulus or internal experience for the required period of time.**<br>*Inclusions: functions of sustaining attention, shifting attention, dividing attention, sharing attention; concentration; distractibility*<br>*Exclusions: consciousness functions (b110); energy and drive functions (b130); sleep functions (b134); memory functions (b144); psychomotor functions (b147); perceptual functions (b156)*
**b152**	**Emotional functions**
	**Specific mental functions related to the feeling and affective components of the processes of the mind.**<br>*Inclusions: functions of appropriateness of emotion, regulation and range of emotion; affect; sadness, happiness, love, fear, anger, hate, tension, anxiety, joy, sorrow; lability of emotion; flattening of affect*<br>*Exclusions: temperament and personality functions (b126); energy and drive functions (b130)*
**b156**	**Perceptual functions**
	**Specific mental functions of recognizing and interpreting sensory stimuli.**<br>*Inclusions: functions of auditory, visual, olfactory, gustatory, tactile and visuospatial perception, such as hallucination or illusion*<br>*Exclusions: consciousness functions (b110); orientation functions (b114); attention functions (b140); memory functions (b144); mental functions of language (b167); seeing and related functions (b210-b229); hearing and vestibular functions (b230-b249); additional sensory functions (b250-b279)*
**b163**	**Basic cognitive functions**
	**Mental functions involved in acquisition of knowledge about objects, events and experiences; and the organization and application of that knowledge in tasks requiring mental activity.**<br>*Inclusion: functions of cognitive development of representation, knowing and reasoning*<br>*Exclusion: higher level cognitive functions (b164)*

**BODY FUNCTIONS**	**(Continued)**
**b164**	**Higher-level cognitive functions**
	**Specific mental functions especially dependent on the frontal lobes of the brain, including complex goal-directed behaviours such as decision-making, abstract thinking, planning and carrying out plans, mental flexibility, and deciding which behaviours are appropriate under what circumstances; often called executive functions.**<br>*Inclusions: functions of abstraction and organization of ideas; time management, insight and judgement; concept formation, categorization and cognitive flexibility*<br>*Exclusions: memory functions (b144); thought functions (b160); mental functions of language (b167); calculation functions (b172)*
**b167**	**Mental functions of language**
	**Specific mental functions of recognizing and using signs, symbols and other components of a language.**<br>*Inclusions: functions of reception and decryption of spoken, written or other forms of language such as sign language; functions of expression of spoken, written or other forms of language; integrative language functions, spoken and written, such as involved in receptive, expressive, Broca's, Wernicke's and conduction aphasia*<br>*Exclusions: attention functions (b140); memory functions (b144); perceptual functions (b156); thought functions (b160); higher-level cognitive functions (b164); calculation functions (b172); mental functions of complex movements (b176); Chapter 2 Sensory Functions and Pain; Chapter 3 Voice and Speech Functions*
**b210**	**Seeing functions**
	**Sensory functions relating to sensing the presence of light and sensing the form, size, shape and colour of the visual stimuli.**<br>*Inclusions: visual acuity functions; visual field functions; quality of vision; functions of sensing light and colour, visual acuity of distant and near vision, monocular and binocular vision; visual picture quality; impairments such as myopia, hypermetropia, astigmatism, hemianopia, colour-blindness, tunnel vision, central and peripheral scotoma, diplopia, night blindness and impaired adaptability to light*<br>*Exclusion: perceptual functions (b156)*
**b2152**	**Functions of external muscles of the eye**
	**Functions of the muscles that are used to look in different directions, to follow an object as it moves across the visual field, to produce saccadic**

BODY FUNCTIONS	(Continued)
	**jumps to catch up with a moving target, and to fix the eye.** *Inclusions: nystagmus; cooperation of both eyes*
**b230**	**Hearing functions**
	**Sensory functions relating to sensing the presence of sounds and discriminating the location, pitch, loudness and quality of sounds.** *Inclusions: functions of hearing, auditory discrimination, localization of sound source, lateralization of sound, speech discrimination; impairments such as deafness, hearing impairment and hearing loss* *Exclusions: perceptual functions (b156) and mental functions of language (b167)*
**b260**	**Proprioceptive function**
	**Sensory functions of sensing the relative position of body parts.** *Inclusions: functions of statesthesia and kinaesthesia* *Exclusions: vestibular functions (b235); sensations related to muscles and movement functions (b780)*
**b280**	**Sensation of pain**
	**Sensation of unpleasant feeling indicating potential or actual damage to some body structure.** *Inclusions: sensations of generalized or localized pain in one or more body part, pain in a dermatome, stabbing pain, burning pain, dull pain, aching pain; impairments such as myalgia, analgesia and hyperalgesia*
**b320**	**Articulation functions**
	**Functions of the production of speech sounds.** *Inclusions: functions of enunciation, articulation of phonemes; spastic, ataxic, flaccid dysarthria; anarthria Exclusions: mental functions of language (b167); voice functions (b310)*
**b440**	**Respiration functions**
	**Functions of inhaling air into the lungs, the exchange of gases between air and blood, and exhaling air.** *Inclusions: functions of respiration rate, rhythm and depth; impairments such as apnoea, hyperventilation, irregular respiration, paradoxical respiration and bronchial spasm and as in pulmonary emphysema; upper pulmonary obstruction, reduction in airflow through upper and lower airways.* *Exclusions: respiratory muscle functions (b445); additional respiratory functions (b450); exercise tolerance functions (b455)*
**b445**	**Respiratory muscle functions**
	**Functions of the muscles involved in breathing.** *Inclusions: functions of thoracic respiratory muscles; functions of the diaphragm; functions of accessory respiratory muscles* *Exclusions: respiration functions (b440); additional respiratory functions (b450); exercise tolerance functions (b455)*
**b4501**	**Transportation of airways mucus**
	**Functions of transporting mucus of upper and lower airways**
**b455**	**Exercise tolerance functions**
	**Functions related to respiratory and cardiovascular capacity as required for enduring physical exertion.** *Inclusions: functions of physical endurance, aerobic capacity, stamina and fatiguability* *Exclusions: functions of the cardiovascular system (b410-b429); haematological system functions (b430); respiration functions (b440); respiratory muscle functions (b445); additional respiratory functions (b450)*
**b510**	**Ingestion functions**
	**Functions related to taking in and manipulating solids or liquids through the mouth into the body.** *Inclusions: functions of sucking, chewing and biting, manipulating food in the mouth, salivation, swallowing, burping, regurgitation, spitting and vomiting; impairments such as dysphagia, aspiration of food, aerophagia, excessive salivation, drooling and insufficient salivation* *Exclusion: sensations associated with digestive system (b535)*
**b525**	**Defecation functions**
	**Functions of elimination of wastes and undigested food as faeces and related functions.** *Inclusions: functions of elimination, faecal consistency, frequency of defecation; faecal continence, flatulence; impairments such as constipation, diarrhoea, watery stool and anal sphincter incompetence or incontinence* *Exclusions: digestive functions (b515); assimilation functions (b520); sensations associated with the digestive system (b535)*
**b530**	**Weight maintenance functions**
	**Functions of maintaining appropriate body weight, including weight gain during the developmental period.** *Inclusions: functions of maintenance of acceptable Body Mass Index (BMI); impairments such as underweight, cachexia, wasting, overweight, emaciation and such as in primary and secondary obesity* *Exclusions: assimilation functions (b520); general metabolic functions (b540); endocrine gland functions (b555)*
**b620**	**Urination functions**
	**Functions of discharge of urine from the urinary bladder.** *Inclusions: functions of urination, frequency of urination, urinary continence; impairments such as in stress, urge, reflex, overflow, continuous incontinence, dribbling, automatic bladder, polyuria, urinary retention and urinary urgency* *Exclusions: urinary excretory functions (b610); sensations associated with urinary functions (b630)*
**b710**	**Mobility of joint functions**

**BODY FUNCTIONS**	**(Continued)**
	**Functions of the range and ease of movement of a joint.**<br>*Inclusions: functions of mobility of single or several joints, vertebral, shoulder, elbow, wrist, hip, knee, ankle, small joints of hands and feet; mobility of joints generalized; impairments such as in hypermobility of joints, frozen joints, frozen shoulder, arthritis*<br>*Exclusions: stability of joint functions (b715); control of voluntary movement functions (b760)*
**b715**	**Stability of joint functions**
	**Functions of the maintenance of structural integrity of the joints.**<br>*Inclusions: functions of the stability of a single joint, several joints, and joints generalized; impairments such as in unstable shoulder joint, dislocation of a joint, dislocation of shoulder and hip*<br>*Exclusion: mobility of joint functions (b710)*
**b730**	**Muscle power functions**
	**Functions related to the force generated by the contraction of a muscle or muscle groups.**<br>*Inclusions: functions associated with the power of specific muscles and muscle groups, muscles of one limb, one side of the body, the lower half of the body, all limbs, the trunk and the body as a whole; impairments such as weakness of small muscles in feet and hands, muscle paresis, muscle paralysis, monoplegia, hemiplegia, paraplegia, quadriplegia and akinetic mutism*<br>*Exclusions: functions of structures adjoining the eye (b215); muscle tone functions (b735); muscle endurance functions (b740)*
**b735**	**Muscle tone functions**
	**Functions related to the tension present in the resting muscles and the resistance offered when trying to move the muscles passively.**<br>*Inclusions: functions associated with the tension of isolated muscles and muscle groups,muscles of one limb, one side of the body and the lower half of the body, muscles of all limbs, muscles of the trunk, and all muscles of the body; impairments such as hypotonia, hypertonia and muscle spasticity, myotonia and paramyotonia*<br>*Exclusions: muscle power functions (b730); muscle endurance functions (b740)*
**b740**	**Muscle endurance functions**
	**Functions related to sustaining muscle contraction for the required period of time.**<br>*Inclusions: functions associated with sustaining muscle contraction for isolated muscles and muscle groups, and all muscles of the body; impairments such as in myasthenia gravis*<br>*Exclusions: exercise tolerance functions (b455); muscle power functions (b730); muscle tone functions (b735)*
**b755**	**Involuntary movement reaction functions**
	**Functions of involuntary contractions of large muscles or the whole body induced by body position, balance and threatening stimuli.**<br>*Inclusions: functions of postural reactions, righting reactions, body adjustment reactions, balance reactions, supporting reactions, defensive reactions*<br>*Exclusion: motor reflex functions (b750)*
**b760**	**Control of voluntary movement functions**
	**Functions associated with control over and coordination of voluntary movements.**<br>*Inclusions: functions of control of simple voluntary movements and of complex voluntary movements, coordination of voluntary movements, supportive functions of arm or leg, right left motor coordination, eye hand coordination, eye foot coordination; impairments such as control and coordination problems, e.g. clumsiness and dysdiadochokinesia*<br>*Exclusions: muscle power functions (b730); involuntary movement functions (b765); gait pattern functions (b770)*
**b765**	**Involuntary movement functions**
	**Functions of unintentional, non- or semi-purposive involuntary contractions of a muscle or group of muscles.**<br>*Inclusions: involuntary contractions of muscles; impairments such as tremors, tics, mannerisms, stereotypies, motor perseveration, chorea, athetosis, vocal tics, dystonic movements and dyskinesia*<br>*Exclusions: control of voluntary movement functions (b760); gait pattern functions (b770)*
**b770**	**Gait pattern functions**
	**Functions of movement patterns associated with walking, running or other whole body movements.**<br>*Inclusions: walking patterns and running patterns; impairments such as spastic gait, hemiplegic gait, paraplegic gait, asymmetric gait, limping and stiff gait pattern*<br>*Exclusions: muscle power functions (b730); muscle tone functions (b735); control of voluntary movement functions (b760); involuntary movement functions (b765)*
**b810**	**Protective functions of the skin**
	**Functions of the skin for protecting the body from physical, chemical and biological threats.**<br>Inclusions: functions of protecting against the sun and other radiation, photosensitivity, pigmentation, quality of skin; insulating function of skin, callus formation, hardening; impairments such as broken skin, ulcers, bedsores and thinning of skin<br>Exclusions: repair functions of the skin (b820); other functions of the skin (b830)

**ACTIVITIES AND PARTICIPATION (N = 58)**<br>= execution of a task or action by an individual and involvement in a life situation	
**d110**	**Watching**
	**Using the sense of seeing intentionally to experience visual stimuli, such as visually tracking an object, watching persons, looking at a sporting event, person, or children playing.**
**d115**	**Listening**
	**Using the sense of hearing intentionally to experience auditory stimuli, such as listening to a radio, the human voice, to music, a lecture, or to a story told.**
**d120**	**Other pursposeful sensing**
	**Using the body's other basic senses intentionally to experience stimuli, such as touching and feeling textures, tasting sweets or smelling flowers.**
**d130**	**Copying**
	**Imitating or mimicking as a basic component of learning, such as copying, repeating a facial expression, a gesture, a sound or the letters of an alphabet.**<br>*Inclusion: immediate imitation of an action or behaviour.*
**d131**	**Learning through actions with objects**
	**Learning through simple actions on a single object, two or more objects, symbolic and pretend play, such as in hitting an object, banging blocks and playing with dolls or cars.**
**d133**	**Acquiring language**
	**Developing the competence to represent persons, objects, events and feelings through words, symbols, phrases and sentences.**<br>*Exclusions: acquiring additional language (d134); communication (d310-d399)*
**d137**	**Acquiring concepts**
	**Developing competence to understand and use basic and complex concepts related to the characteristics of things, persons or events**
**d140**	**Learning to read**
	**Developing the competence to read written material (including Braille and other symbols) with fluency and accuracy, such as recognizing characters and alphabets, sounding out words written words with correct pronunciation, and understanding words and phrases.**
**d145**	**Learning to write**
	**Developing the competence to produce symbols that represent sounds, words or phrases in order to convey meaning (including Braille writing and other symbols), such as spelling effectively and using correct grammar.**
**d155**	**Acquiring skills**
	**Developing basic and complex competencies in integrated sets of actions or tasks so as to initiate and follow through with the acquisition of a skill, such as manipulating tools or toys, or playing games.**<br>*Inclusions: acquiring basic and complex skills*<br>*Exclusions: learning to write (d145) and writing (d170), learning to play (d131)*
**d160**	**Focusing attention**
	**Intentionally focusing on specific stimuli, such as by filtering out distracting noises.**
**d166**	**Reading**
	**Performing activities involved in the comprehension and interpretation of written language (e.g. books, instructions, newspapers in text or Braille), for the purpose of obtaining general knowledge or specific information.**<br>*Inclusion: Comprehension and interpretation of written language in standard form of letters or characters as well as text created with unique symbols such as icons*<br>*Exclusion: learning to read (d140).*
**d170**	**Writing**
	**Using or producing symbols or language to convey information, such as producing a written record of events or ideas or drafting a letter.**<br>*Exclusion: learning to write (d145)*
**d172**	**Calculating**
	**Performing computations by applying mathematical principles to solve problems that are described in words and producing or displaying the results, such as computing the sum of three numbers or finding the result of dividing one number by another.**<br>*Exclusion: learning to calculate (d150)*

ACTIVITIES AND PARTICIPATION	(Continued)
**d175**	**Solving problems**
	**Finding solutions to questions or situations by identifying and analysing issues, developing options and solutions, evaluating potential effects of solutions, and executing a chosen solution, such as in resolving a dispute between two people.**<br>*Inclusions: solving simple and complex problems*<br>*Exclusions: thinking (d163); making decisions (d177)*
**d177**	**Making decisions**
	**Making a choice among options, implementing the choice, and evaluating the effects of the choice, such as selecting and purchasing a specific item, or deciding to undertake and undertaking one task from among several tasks that need to be done.**<br>*Exclusions: thinking (d163); solving problems (d175)*
**d220**	**Undertaking multiple tasks**
	**Carrying out simple or complex and coordinated actions as components of multiple, integrated and complex tasks in sequence or simultaneously.**<br>*Inclusions: undertaking multiple tasks; completing multiple tasks; undertaking multiple tasks independently and in a group*<br>*Exclusions: acquiring skills (d155); solving problems (d175); making decisions (d177); undertaking a single task (d210)*
**d230**	**Carrying out daily routine**
	**Carrying out simple or complex and coordinated actions in order to plan, manage and complete the requirements of day-to-day procedures or duties, such as budgeting time and making plans for separate activities throughout the day.**<br>*Inclusions: managing and completing the daily routine; managing one's own activity level*<br>*Exclusion: undertaking multiple tasks (d220)*
**d250**	**Managing one's own behaviour**
	**Carrying out simple or complex and coordinated actions in a consistent manner in response to new situations, persons or experiences, such as being quiet in a library.**
**d310**	**Communicating with - receiving - spoken messages**
	**Comprehending literal and implied meanings of messages in spoken language, such as understanding that a statement asserts a fact or is an idiomatic expression, such as responding and comprehending spoken messages.**
**d330**	**Speaking**
	**Producing words, phrases and longer passages in spoken messages with literal and implied meaning, such as expressing a fact or telling a story in oral language**
**d331**	**Pre-talking**
	**Vocalizing when aware of another person in the proximal environment, such as producing sounds when the mother is close; babbling; babbling in turn-taking activities. Vocalizing in response to speech through imitating speech-sounds in a turn taking procedure.**
**d335**	**Producing non-verbal messages**
	**Using gestures, symbols and drawings to convey messages, such as shaking one's head to indicate disagreement or drawing a picture or diagram to convey a fact or complex idea.**<br>*Inclusions: producing body gestures, signs, symbols, drawings and photographs*
**d350**	**Conversation**
	**Starting, sustaining and ending an interchange of thoughts and ideas, carried out by means of spoken, written, sign or other forms of language, with one or more persons one knows or who are strangers, in formal or casual settings.**<br>*Inclusions: starting, sustaining and ending a conversation; conversing with one or many people.*
**d360**	**Using communication devices and techniques**
	**Using devices, techniques and other means for the purposes of communicating, such as calling a friend on the telephone.**<br>*Inclusions: using telecommunication devices, using writing machines and communication techniques*
**d410**	**Changing basic body position**
	**Getting into and out of a body position and moving from one location to another,such as rolling from one side to the other, sitting, standing, getting up out of a chair to lie down on a bed, and getting into and out of positions of kneeling or squatting.**<br>*Inclusion: changing body position from lying down, from squatting or kneeling, from sitting or standing, bending and shifting the body's centre of gravity*<br>*Exclusion: transferring oneself (d420)*
**d415**	**Maintaining a body position**
	**Staying in the same body position as required, such as remaining seated or remaining standing for work or school.**<br>*Inclusions: maintaining a lying, squatting, kneeling, sitting and standing position*

ACTIVITIES AND PARTICIPATION	(Continued)
d420	Transferring oneself
	**Moving from one surface to another, such as sliding along a bench or moving from a bed to a chair, without changing body position.**<br>*Inclusion: transferring oneself while sitting or lying*<br>*Exclusion: changing basic body position (d410)*
d430	Lifting and carrying objects
	**Raising up an object or taking something from one place to another, such as when lifting a cup or toy, or carrying a box or child from one room to another.**<br>*Inclusions: lifting, carrying in the hands or arms, or on shoulders, hip, back or head; putting down*
d435	Moving objects with lower extremities
	**Performing coordinated actions aimed at moving an object by using the legs and feet, such as kicking a ball or pushing pedals on a bicycle.**<br>*Inclusions: pushing with lower extremities; kicking*
d440	Fine hand use
	**Performing the coordinated actions of handling objects, picking up, manipulating and releasing them using one's hand, fingers and thumb, such as required to lift coins off a table or turn a dial or knob.**<br>*Inclusions: picking up, grasping, manipulating and releasing*
d445	Hand and arm use
	**Performing the coordinated actions required to move objects or to manipulate them by using hands and arms, such as when turning door handles or throwing or catching an object**<br>*Inclusions: pulling or pushing objects; reaching; turning or twisting the hands or arms; throwing; catching*<br>*Exclusion: fine hand use (d440)*
d450	Walking
	**Moving along a surface on foot, step by step, so that one foot is always on the ground, such as when strolling, sauntering, walking forwards, backwards or sideways.**<br>*Inclusions: walking short or long distances; walking on different surfaces; walking around obstacles*<br>*Exclusions: transferring oneself (d420); moving around (d455)*
d455	Moving around
	**Moving the whole body from one place to another by means other than walking, such as climbing over a rock or running down a street, skipping, scampering, jumping, somersaulting or running around obstacles.**<br>*Inclusions: crawling, climbing, running, jogging, jumping and swimming, scooting, rolling and schuffling*<br>*Exclusions: transferring oneself (d420); walking (d450)*
d460	Moving around in different locations
	**Walking and moving around in various places and situations, such as walking between rooms in a house, within a building, or down the street of a town.**<br>*Inclusions: moving around within the home, crawling or climbing within the home; walking or moving within buildings other than the home, and outside the home and other buildings*
d465	Moving around using equipment
	**Moving the whole body from place to place, on any surface or space, by using specific devices designed to facilitate moving or create other ways of moving around, such as with skates, skis, scuba equipment, swim fins, or moving down the street in a wheelchair or a walker.**<br>*Exclusions: transferring oneself (d420); walking (d450); moving around (d455); using transportation (d470); driving (d475)*
d470	Using transportation
	**Using transportation to move around as a passenger, such as being driven in a car, bus, rickshaw, jitney, pram or stroller, animal-powered vehicle, private or public taxi, train, tram, subway, boat or aircraft.**<br>*Inclusions: using human-powered transportation; using private motorized or public transportation*<br>*Exclusions: moving around using equipment (d465); driving (d475)*
d510	Washing oneself
	**Washing and drying one's whole body, or body parts, using water and appropriate cleaning and drying materials or methods, such as bathing, showering, washing hands and feet, face and hair, and drying with a towel.**<br>*Inclusions: washing body parts, the whole body; and drying oneself*<br>*Exclusions: caring for body parts (d520); toileting (d530)*
d520	Caring for body parts
	**Looking after those parts of the body, such as skin, face, teeth, scalp, nails and genitals, that require more than washing and drying.**<br>*Inclusions: caring for skin, teeth, hair, finger and toe nails, and nose*<br>*Exclusions: washing oneself (d510); toileting (d530)*
d530	Toileting
	**Indicating the need for, planning and carrying out the elimination of human waste (menstruation, urination and defecation), and cleaning oneself afterwards.**<br>*Inclusions: regulating urination, defecation and menstrual care*

ACTIVITIES AND PARTICIPATION	(Continued)
	Exclusions: washing oneself (d510); caring for body parts (d520)
d540	Dressing
	**Carrying out the coordinated actions and tasks of putting on and taking off clothes and footwear in sequence and in keeping with climatic and social conditions, such as by putting on, adjusting and removing shirts, skirts, blouses, pants, undergarments, saris, kimono, tights, hats, gloves, coats, shoes, boots, sandals and slippers.**<br>*Inclusions: putting on or taking off clothes and footwear and choosing appropriate clothing*
d550	Eating
	**Indicating need for, and carrying out the coordinated tasks and actions of eating food that has been served, bringing it to the mouth and consuming it in culturally acceptable ways, cutting or breaking food into pieces, opening bottles and cans, using eating implements, having meals, feasting or dining.**<br>*Exclusion: drinking (d560)*
d560	Drinking
	**Indicating need for, and taking hold of a drink, bringing it to the mouth and consuming the drink in culturally acceptable ways; mixing, stirring and pouring liquids for drinking, opening bottles and cans, drinking through a straw or drinking running water, such as from a tap or a spring; feeding from the breast.**<br>*Exclusion: eating (d550)*
d570	Looking after one's health
	**Ensuring or indicating needs about physical comfort, health and physical and mental well-being, such as by maintaining a balanced diet and an appropriate level of physical activity, keeping warm or cool, avoiding harm to health, following safe sex practices, including using condoms, getting immunizations and regular physical examinations.**<br>*Inclusions: ensuring one's physical comfort; managing diet and fitness; maintaining one's health*
d630	Preparing meals
	**Planning, organizing, cooking and serving simple and complex meals for oneself and others, such as by making a menu, selecting edible food and drink, getting together ingredients for preparing meals, cooking with heat and preparing cold foods and drinks, and serving the food.**<br>*Inclusions: preparing simple and complex meals*<br>*Exclusions: eating (d550); drinking (d560); acquisition of goods and services (d620); doing housework (d640); caring for household objects (d650); caring for others (d660)*
d640	Doing housework
	**Managing a household by cleaning the house, washing clothes, using household appliances, storing food and disposing of garbage, such as by sweeping, mopping, washing counters, walls and other surfaces; collecting and disposing of household garbage; tidying rooms, closets and drawers; collecting, washing, drying, folding and ironing clothes; cleaning footwear; using brooms, brushes and vacuum cleaners; using washing machines, driers and irons.**<br>*Inclusions: washing and drying clothes and garments; cleaning cooking area and utensils; cleaning living area; using household appliances, storing daily necessities and disposing of garbage*<br>*Exclusions: acquiring a place to live (d610); acquisition of goods and services (d620); preparing meals (d630); caring for household objects (d650); caring for others (d660)*
d710	Basic interpersonal interactions
	**Interacting with people in a contextually and socially appropriate manner, such as by showing consideration and esteem when appropriate, or responding to the feelings of others.**<br>*Inclusions: showing respect, warmth, appreciation, and tolerance in relationships; responding to criticism and social cues in relationships; and using appropriate physical contact in relationships*
d720	Complex interpersonal interactions
	**Maintaining and managing interactions with other people, in a contextually and socially appropriate manner, such as by regulating emotions and impulses, controlling verbal and physical aggression, acting independently in social interactions, and acting in accordance with social rules and conventions.**<br>*Inclusions: playing with others, forming and terminating relationships; regulating behaviours within interactions; interacting according to social rules; and maintaining social space*
d750	Informal social relationships
	**Entering into relationships with others, such as casual relationships with people living in the same community or residence, or with co-workers, students, playmates or people with similar backgrounds or professions.**<br>*Inclusions: informal relationships with friends, neighbours, acquaintances, co-inhabitants and peers*

ACTIVITIES AND PARTICIPATION (Continued)	
**d760**	**Family relationships**
	Creating and maintaining kinship relationships, such as with members of the nuclear family, extended family, foster and adopted family and step-relationships, more distant relationships such as second cousins, or legal guardians.<br>*Inclusions: parent-child and child-parent relationships, sibling and extended family relationships*
**d770**	**Intimate relationships**
	Creating and maintaining close or romantic relationships between individuals, such as husband and wife, lovers or sexual partners.<br>*Inclusions: romantic, spousal and sexual relationships*
**d815**	**Preschool education**
	Learning at an initial level of organized instruction in the home or in the community designed primarily to introduce a child to a school-type environment and prepare the child for compulsory education, such as by acquiring skills in a day-care or similar setting in preparation for school (e.g. educational services provided in the home or in community settings designed to promote health and cognitive, motor, language and social development and readiness skills for formal education).
**d820**	**School education**
	Gaining admission to school, education; engaging in all school-related responsibilities and privileges; learning the course material, subjects and other curriculum requirements in a primary or secondary education programme, including attending school regularly; working cooperatively with other students, taking direction from teachers, organizing, studying and completing assigned tasks and projects, and advancing to other stages of education.
**d845**	**Acquiring, keeping and terminating a job**
	Seeking, finding and choosing employment, being hired and accepting employment, maintaining and advancing through a job, trade, occupation or profession, and leaving a job in an appropriate manner.<br>*Inclusions: seeking employment; preparing a resume or curriculum vitae; contacting employers and preparing interviews; maintaining a job; monitoring one's own work performance; giving notice; and terminating a job*
**d860**	**Basic economic transactions**
	Engaging in any form of simple economic transaction, such as using money to purchase food or bartering, exchanging goods or services; or saving money.
**d880**	**Engagement in play**
	Purposeful, sustained engagement in activities with objects, toys, materials or games, occupying oneself or with others.
**d910**	**Community life**
	Engaging in all aspects of community social life, such as engaging in charitable organizations, service clubs or professional social organizations.<br>*Inclusions: informal and formal associations; ceremonies*<br>*Exclusions: non-remunerative employment (d855); recreation and leisure (d920); religion and spirituality (d930); political life and citizenship (d950)*
**d920**	**Recreation and leisure**
	Engaging in any form of play, recreational or leisure activity, such as informal or organized play and sports, programmes of physical fitness, relaxation, amusement or diversion, going to art galleries, museums, cinemas or theatres; engaging in crafts or hobbies, reading for enjoyment, playing musical instruments; sightseeing, tourism and travelling for pleasure.<br>*Inclusions: games, sports, arts and culture, crafts, hobbies and socializing*<br>*Exclusions: riding animals for transportation (d480); remunerative and nonremunerative work (d850 and d855); engagement in play (d880); religion and spirituality (d930); political life and citizenship (d950)*

ENVIRONMENTAL FACTORS (N = 36)<br>= make up the physical, social and attitudinal environment in which people live and conduct their lives	
**e110**	**Products or substances for personal consumption**
	Any natural or human-made object or substance gathered, processed or manufactured for ingestion.<br>*Inclusions: food (including breast milk), drink and drugs*
**e115**	**Products and technology for personal use in daily living**
	Equipment, products and technologies used by people in daily activities, including those adapted or specially designed, located in, on or near the person using them.<br>*Inclusions: general and assistive products and technology for personal use*<br>*Exclusions: products and technology for personal indoor and outdoor mobility and transportation (e120); products and technology for communication (e125)*
**e120**	**Products and technology for personal indoor and outdoor mobility and transportation**

	Equipment, products and technologies used by people in activities of moving inside and outside buildings, including those adapted or specially designed, located in, on or near the person using them.<br>*Inclusions: general and assistive products and technology for personal indoor and outdoor mobility and transportation*
e125	Products and technology for communication
	Equipment, products and technologies used by people in activities of sending and receiving information, including those adapted or specially designed, located in, on or near the person using them.<br>*Inclusions: general and assistive products and technology for communication*
e130	Products and technology for education
	Equipment, products, processes, methods and technology used for acquisition of knowledge, expertise or skill, including those adapted or specially designed.<br>*Inclusion: general and assistive products and technology for education*
e140	Products and technology for culture, recreation and sport
	Equipment, products and technology used for the conduct and enhancement of cultural, recreational and sporting activities, including those adapted or specially designed.<br>*Inclusion: general and assistive products and technology for culture, recreation and sport*<br>*Exclusion: products and technology for play (e1152)*
e150	Design, construction and building products and technology of buildings for public use
	Products and technology that constitute an individual's indoor and outdoor human-made environment that is planned, designed and constructed for public use, including those adapted or specially designed.<br>*Inclusions: design, construction and building products and technology of entrances and exits, facilities and routing*
e155	Design, construction and building products and technology of buildings for private use
	Products and technology that constitute an individual's indoor and outdoor human-made environment that is planned, designed and constructed for private use (e.g. home, dwelling), including those adapted or specially designed.<br>*Inclusions: design, construction and building products and technology of entrances and exits, facilities and routing*
e160	Products and technology of land development
	Products and technology of land areas, as they affect an individual's outdoor environment through the implementation of land use policies, design, planning and development of space, including those adapted or specially designed.<br>*Inclusions: products and technology of land areas that have been organized by the implementation of land use policies, such as rural areas, suburban areas, urban areas, parks, conservation areas and wildlife reserves*
e165	Assets
	Products or objects of economic exchange such as money, goods, property and other valuables that an individual owns or of which he or she has rights of use or rights of benefit, such as child support payment or wills for children or dependent persons.<br>*Inclusions: tangible and intangible products and goods, financial assets*
ENVIRONMENTAL FACTORS (Continued)	
e310	Immediate family
	Individuals related by birth, marriage or other relationship recognized by the culture as immediate family, such as spouses, partners, parents, siblings, children, foster parents, adoptive parents and grandparents.<br>*Exclusions: extended family (e315); personal care providers and personal assistants (e340)*
e315	Extended family
	Individuals related through family or marriage or other relationships recognized by the culture as extended family, such as aunts, uncles, nephews and nieces.<br>*Exclusion: immediate family (e310)*
e320	Friends
	Individuals who are close and ongoing participants in relationships characterized by trust and mutual support.
e325	Acquaintances, peers, colleagues, neighbours and community members
	Individuals who are familiar to each other as acquaintances, peers, colleagues, neighbours, and community members, in situations of work, school, recreation, or other aspects of life, and who share demographic features such as age, gender, religious creed or ethnicity or pursue common interests.<br>*Exclusions: associations and organizational services (e5550)*
e330	People in position of authority
	Individuals who have decision-making responsibilities for others and who have socially defined influence or power based on their social, economic, cultural or religious roles in society, such as teachers, employers, supervisors, religious leaders, substitute decision-makers, guardians or trustees.
e340	Personal care providers and personal assistants
	Individuals who provide services as required to support individuals in their daily activities and maintenance of performance at work, education or other life situation, provided either through public or private funds, or else on a voluntary basis, such as providers of support for home-making and maintenance, personal assistants, transport assistants, paid help, nannies and others who function as primary caregivers.

	Exclusions: immediate family (e310); extended family (e315); friends (e320); general social support services (e5750); health professionals (e355)
e355	Health professionals
	All service providers working within the context of the health system, such as doctors, nurses, physiotherapists, occupational therapists, speech therapists, audiologists, orthotist-prosthetists, medical social workers.<br>*Exclusion: other professionals (e360)*
e410	Individual attitudes of immediate family members
	General or specific opinions and beliefs of immediate family members about the person or about other matters (e.g. social, political and economic issues), that influence individual behaviour and actions.
e415	Individual attitudes of extended family members
	General or specific opinions and beliefs of extended family members about the person or about other matters (e.g. social, political and economic issues), that influence individual behaviour and actions.
e420	Individual attitude of friends
	General or specific opinions and beliefs of friends about the person or about other matters (e.g. social, political and economic issues), that influence individual behaviour and actions.
e425	Individual attitudes of acquaintances, peers, colleagues, neighbours and community members
	General or specific opinions and beliefs of acquaintances, peers, colleagues, neighbours and community members about the person or about other matters (e.g. social, political and economic issues), that influence individual behaviour and actions.
e430	Individual attitudes of people in position of authority
	General or specific opinions and beliefs of people in positions of authority about the person or about other matters (e.g. social, political and economic issues), that influence individual behaviour and actions.
e440	Individual attitudes of personal care providers and personal assistants
	General or specific opinions and beliefs of personal care providers and personal assistants about the person or about other matters (e.g. social, political and economic issues), that influence individual behaviour and actions.
e450	Individual attitudes of health professionals
	General or specific opinions and beliefs of health professionals about the person or about other matters (e.g. social, political and economic issues), that influence individual behaviour and actions.
e460	Societal attitudes
	General or specific opinions and beliefs generally held by people of a culture, society, subcultural or other social group about other individuals or about other social, political and economic issues, that influence group or individual behaviour and actions.
e465	Social norms, practices and ideologies
	Customs, practices, rules and abstract systems of values and normative beliefs (e.g. ideologies, normative world views and moral philosophies) that arise within social contexts and that affect or create societal and individual practices and behaviours, such as social norms of moral and religious behaviour or etiquette; religious doctrine and resulting norms and practices; norms governing rituals or social gatherings.
e525	Housing services, systems and policies
	Services, systems and policies for the provision of shelters, dwellings or lodging for people.
e540	Transportation services, systems and policies
	Services, systems and policies for enabling people or goods to move or be moved from one location to another.
e550	Legal services, systems and policies
	Services, systems and policies concerning the legislation and other law of a country.
ENVIRONMENTAL FACTORS (Continued)	
e555	Associations and organizational services, systems and policies
	Services, systems and policies relating to groups of people who have joined together in the pursuit of common, noncommercial interests, often with an associated membership structure.
e560	Media services, systems and policies
	Services, systems and policies for the provision of mass communication through radio, television, newspapers and internet.
e570	Social security services, systems and policies
	Services, systems and policies aimed at providing income support to people who, because of age, poverty, unemployment, health condition or disability, require public assistance that is funded either by general tax revenues or contributory schemes.<br>*Exclusion: economic services, systems and policies (e565)*
e575	General social support services, systems and policies

	**Services, systems and policies aimed at providing support to those requiring assistance in areas such as shopping, housework, transport, child care, respite care, self-care and care of others, in order to function more fully in society.** *Exclusions: social security services, systems and policies (e570); personal care providers and personal assistants (e340); health services, systems and policies (e580)*
e580	**Health services, systems and policies**
	**Services, systems and policies for preventing and treating health problems, providing medical rehabilitation and promoting a healthy lifestyle.** *Exclusion: general social support services, systems and policies (e575)*
e585	**Education and training services, systems and policies**
	**Services, systems and policies for the acquisition, maintenance and improvement of knowledge, expertise and vocational or artistic skills. See UNESCO's International Standard Classification of Education (ISCED-1997).**
e590	**Labour and employment services, systems and policies**
	**Services, systems and policies related to finding suitable work for persons who are unemployed or looking for different work, or to support individuals already employed who are seeking promotion.** *Exclusion: economic services, systems and policies (e565)*

Table S2 - Examples of tools, examinations, and questionnaires used to apply the Brief Common ICF Core Set for CP in Russia

ICF	Descriptor	Tools and questionnaires	Others
**b117**	Intellectual functions	Clinical-psychopathological method, Wechsler Intelligence Scale for Children (WISC)	
**b167**	Mental functions of language	Wechsler Intelligence Scale for Children (WISC)	
**b210**	Seeing functions	Does the child have problems seeing?<br>0-1 year: *sensing light, following objects*<br>1-3 years: Avetisov method, the patent №2008120086 of 20.05.2008<br>3-7 years: Orlova tables, Polyak optotypes, the patent №2008120086 of 20.05.2008, *sensing colour*<br>Over 7 years: Orlova, Golovin-Sictsev tables, Polyak optotypes, the patent №2008120086 of 20.05.2008, determination of the central and peripheral vision on the Karl Zeis perimeter or the Ferster hand perimeter, the Donders approximate-control method	Tests and observation by a caregiver, questions to the child over 3 years old
**b280**	Sensation of pain	Wong-Baker Faces Pain Scale for Children 3 Years or Older<br>Wong D. L,, Baker C. M., 1988<br>Kindliche unbehagens-und Schmerzskala (KUSS)<br>Büttner W. et al., 1998<br>http://ilive.com.ua/health/shkala-ocenki-boli-u-detey_105701i15989.html	Questionnaire applied to the mother or to a child in the form of an interview

ICF	Descriptor	Tools and questionnaires	Others
**d360**	Using communication devices and techniques	Using devices for the purposes of communicating (telephone or iPad)<br>Social Communication Questionnaire (SCQ)<br>Rutter M, Bailey A, Lord C. The Social Communication Questionnaire (SCQ), second printing. Western Psychological Services; 2007.	Observation by a caregiver, questionnaire applied to the mother in the form of an interview
**d410**	Changing basic body position	Can the child stand up from a chair?<br>Can the child come to another chair?<br>Can the child lie on a bed?<br>Social Communication Questionnaire (SCQ)<br>Rutter M, Bailey A, Lord C. The Social Communication Questionnaire (SCQ), second printing. Western Psychological Services; 2007	Observation by a caregiver, non-applicable under 2 years old
**d415**	Maintaining a body position	Can the child stand for 5 min.?<br>Can the child remain seated for 5 min.?<br>Social Communication Questionnaire (SCQ)<br>Rutter M, Bailey A, Lord C. The Social Communication Questionnaire (SCQ), second printing. Western Psychological Services; 2007	Observation by a caregiver
**d440**	Fine hand use	Diagnostic tests (drawing, manipulating and releasing objects as a pencil etc)<br>The Manual Ability Classification System (MACS)	Observation by a caregiver
**d445**	Hand and arm use	Performing the coordinated actions required to move objects or to manipulate them:<br>How does the child toss up and catch the ball?<br>How does the child turn a knob?<br>The Manual Ability Classification System (MACS)	Observation by a caregiver

ICF	Descriptor	Tools and questionnaires	Others
**e130**	Products and technology for education	How often the child use equipment, products, methods and technology used for acquisition of knowledge, expertise or skill, including general those or adapted or specially designed? Can the child use them himself/herself or needs help?	Questionnaire applied to the mother or the child in the form of an interview
**e140**	Products and technology for culture, recreation and sport	How often the child use equipment, products and technology used for the conduct and enhancement of cultural, recreational and sporting activities, including general those adapted or specially designed? Can the child use them himself/herself or needs help?	Questionnaire applied to the mother or the child in the form of an interview

Table S3. Distribution of ICF qualifiers entire Brazilian sample with congenital ZIKA virus

Relative frequency of children with microcephaly by ZIKV in body functions, body structures, and activities/participation categories of the Brief Common ICF (International Classification of Functioning Disability and Health) Core Set for cerebral palsy (CP) (n = 34).

International Journal of
Environmental Research and Public Health

MDPI

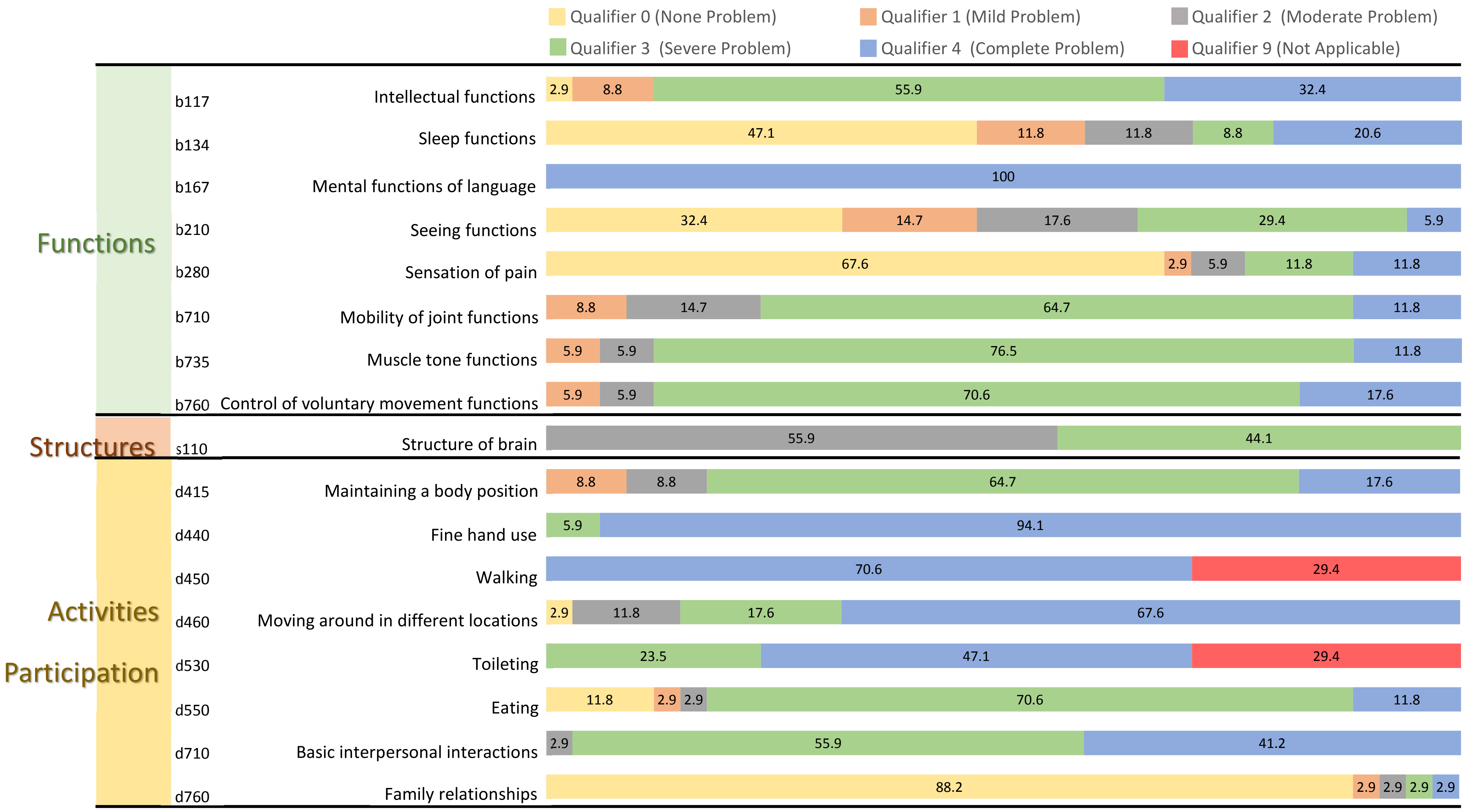

Table S4 Relative frequency of children with microcephaly by ZIKV in environmental factors categories of the Brief Common ICF Core Set for CP (*n* = 34).

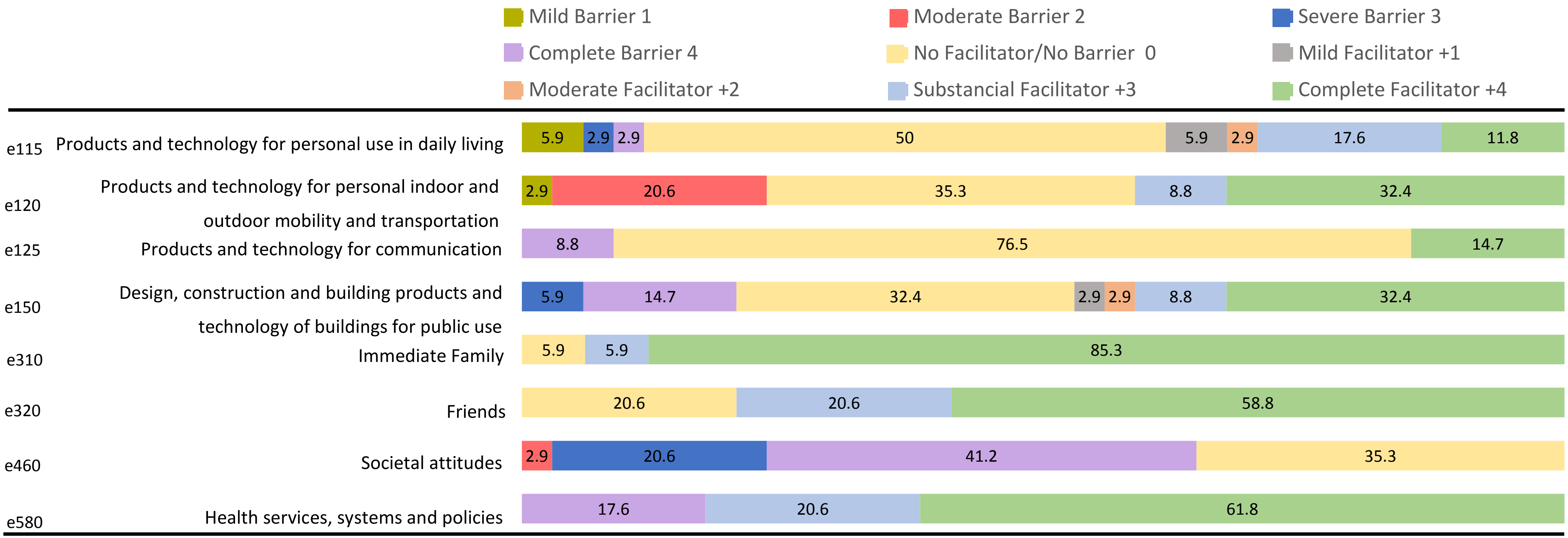

Table S5. Profile of functioning of Malawi children with CP, n=18

Distribution of ICF qualifiers in a community-based rehabilitation in Malawi. ICF qualifiers were rated as: in body functions and activities and participation (Problem/limitations or strengths); in environmental factors (barriers or facilitators)

Profile of functioning children with CP in Malawi		
Body Functions	*Number of children*	*ICF qualifiers Problem/limitation vs Strength*
b117	***NA***	***NA***
b134	***NA***	***NA***
b167	***NA***	***NA***
b210	***NA***	***NA***
b280	***NA***	***NA***
b710	***N=17***	***Problem***
b735	***N=17***	***Problem***
b760	***N=15***	***Problem***
Body Structures		
s110	***NA***	***NA***
Activities and Participation		
d415	***N=10***	***Problem/limitation***
d440	***N=12***	***Strength***
d450	***N=11***	***Strength***
d460	***N=9***	***Strength***
d530	***N=10***	***Problem/limitation***
d550	***N=10***	***Strength***
d710	***N=10***	***Strength***
d760	***NA***	***NA***
Environmental Factors	***Number of children***	***ICF qualifier Barrier vs Facilitator***
e115	***N=7***	***Barrier***
e120	***All***	***Facilitator***
e125	***N=10***	***Barrier***
e150	***N=5***	***Barrier***
e310	***All***	***Facilitator***
e320	***All***	***Facilitator***
e460	***NA***	***NA***
e580	***All***	***Barrier***

NA; not available – information was not available to rate this category

Table S6. Interview guideline for ongoing ZIKA COS study – based on ICF Core Sets for CP

Interview guideline for ongoing study – adapted from Schiariti et al [14] ' He does Not See Himself as being Different': The Perspectives of Children and Caregivers on Relevant Areas of Functioning in Cerebral Palsy. Dev. Med. Child Neurol. 2014, *56*, 853-861.

Questions	ICF Categories
**1.** If you think about your child as an individual, what **personal characteristics** are important about him/her? (Moderator provide an example)	**PERSONAL FACTORS**
2. If you think about the **daily activities**, (Moderator provide an example, show pictures of activities)<br>a. What activities your child is <u>able to do</u>?<br>b. What activities does your child find <u>hard/difficult to do</u>?	**ACTIVITIES & PARTICIPATION**
3. If you think about the **physical and social environment** (**family, school, and neighborhood)** of your child, (Moderator provide an example, show pictures, ask HOME, SCHOOL, COMMUNITY)<br>a. What do you find **helpful or supportive** in facilitating participation in different activities?<br>b. What **challenges or barriers** does your child experience?	**ENVIRONMENTAL FACTORS**
4. If you think about the **body** of your child, which body parts cause your child some difficulties, if any? (Moderator provide an example; may be a picture of the body and they can mark areas with an X)	**BODY STRUCTURES**

5. If you think about the **body** of your child, what parts of his/her body do not work the way it supposes to, if any? (Moderator provide an example, may be a list from the ICF Core Sets then they can mark functions from the list)

BODY FUNCTIONS

6. Tell me about the **biggest problems** for you at the moment.

7. If people were looking into treatments for your child, what are the things you might **hope to improve:**

    a. In terms of their **daily activities?**

    b. In terms of making it **easier for your child to participate** in daily activities (in the neighborhood, at home with the family and at school or nursery, if they attend)?

    c.

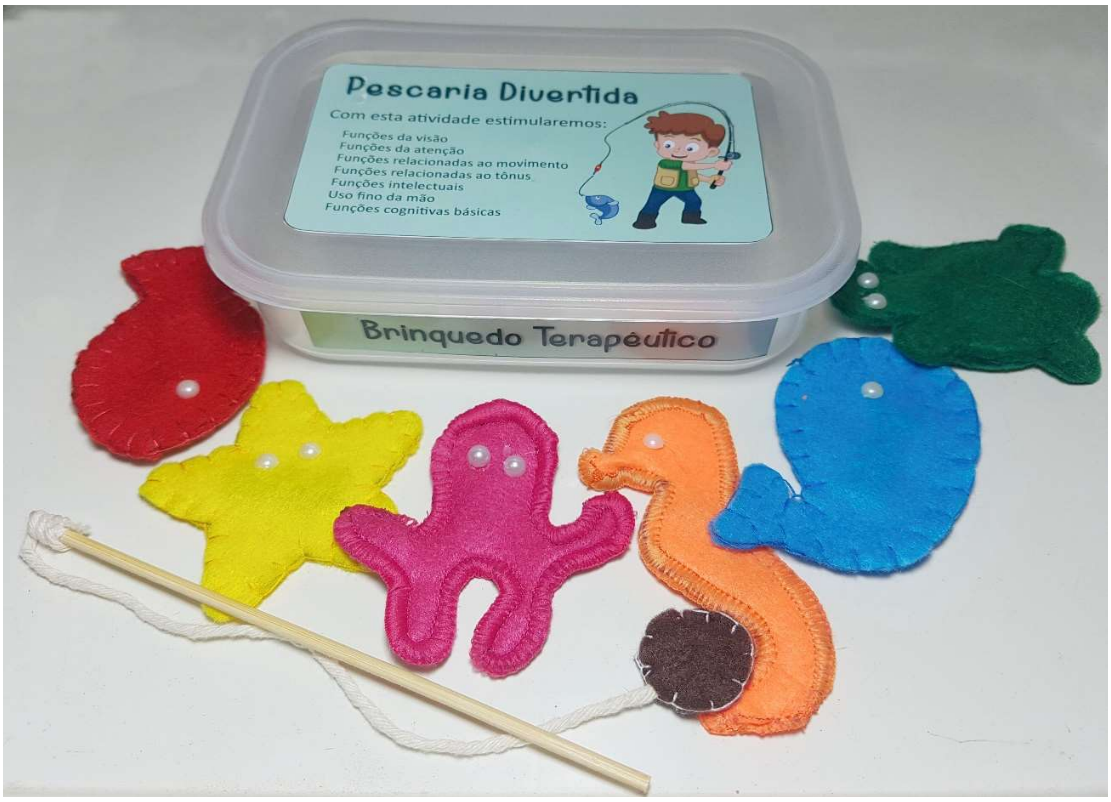

Figure S1. Illustration of early intervention package for participants of ZIKA COS study – based on the ICF model - Brazilian initiative

In gratitude for attendance at focus groups, parents will receive a hand-made educational toy "fun fishery" to stimulate their children at home.
